# Dysregulated *IER3* Expression is Associated with Enhanced Apoptosis in Titin-Based Dilated Cardiomyopathy

**DOI:** 10.3390/ijms18040723

**Published:** 2017-03-29

**Authors:** Qifeng Zhou, Julia Kelley Hahn, Balram Neupane, Parwez Aidery, Siegfried Labeit, Meinrad Gawaz, Michael Gramlich

**Affiliations:** 1 Department of Cardiology and Cardiovascular Diseases, Eberhard Karls University, 72076 Tübingen, Germany; zhouqf09@gmail.com (Q.Z.); hahnjk@gmail.com (J.K.H.); Balram.Neupane@med.uni-tuebingen.de (B.N.); parwez.aidery@med.uni-tuebingen.de (P.A.); meinrad.gawaz@med.uni-tuebingen.de (M.G.); 2 Institute for Integrative Pathophysiology, Universitätsmedizin Mannheim, 68167 Mannheim, Germany; labeit@medma.de

**Keywords:** titin, dilated cardiomyopathy, apoptosis, heart failure, IER3, mouse model

## Abstract

Apoptosis (type I programmed cell death) of cardiomyocytes is a major process that plays a role in the progression of heart failure. The early response gene *IER3* regulates apoptosis in a wide variety of cells and organs. However, its role in heart failure is largely unknown. Here, we investigate the role of *IER3* in an inducible heart failure mouse model. Heart failure was induced in a mouse model that imitates a human titin truncation mutation we found in a patient with dilated cardiomyopathy (DCM). Transferase dUTP nick end labeling (TUNEL) and ssDNA stainings showed induction of apoptosis in titin-deficient cardiomyocytes during heart failure development, while *IER3* response was dysregulated. Chromatin immunoprecipitation and knock-down experiments revealed that IER3 proteins target the promotors of anti-apoptotic genes and act as an anti-apoptotic factor in cardiomyocytes. Its expression is blunted during heart failure development in a titin-deficient mouse model. Targeting the *IER3* pathway to reduce cardiac apoptosis might be an effective therapeutic strategy to combat heart failure.

## 1. Introduction

Congestive heart failure is a major health problem in the western world and affects about 5.7 million adults in the United States [1]. Dilated cardiomyopathy (DCM), which is characterized by a dilatation of the ventricles and impaired left-ventricular function, represents the most common cause of non-ischemic forms of heart failure [2]. DCM is a very heterogeneous disease; around 50 genes involved in various cellular functions have been linked to familial DCM [3]. However, recent high-throughput based studies revealed that mutations in the giant sarcomeric protein titin (also known as connectin) are the predominant genetic cause for DCM and account for 25% of familial forms of DCM [4].

Titin (TTN) is the largest known protein and is mainly expressed in muscular tissue [5]. A single titin molecule spans the entire half sarcomere and links the Z-disc with the M-line. As a pivotal building block of the sarcomere, TTN contributes to the passive force in muscle, and scaffolds in muscle, and also coordinates structural and signaling proteins [6].

Mutations in *TTN* were reported more than a decade ago, when we identified a 2 bp *TTN* insertion mutation in a family with autosomal dominant inherited DCM [6]. This mutation disrupts the reading frame and leads to a premature stop codon in the TTN A-band. To investigate mechanisms that are involved in the pathogenesis of heart failure, we generated a knock-in mouse model that integrates the human insertion mutation in the mouse genome. While the homozygous transgenic embryos die in utero due to severe defects in myocardial development, heterozygous (HET) mice are asymptomatic under resting conditions but develop a DCM-like phenotype under cardiac stress [7].

Apoptosis is a hallmark of the failing human heart [8] and mainly contributes to the cardiac remodeling process in heart failure [9]. Animal studies revealed that forced induction of apoptosis in the mouse heart leads to DCM [10,11]. Accordingly, a caspase-8 transgenic mouse model that develops heart failure can be rescued by inhibition of apoptosis [10].

*IER3*, formerly known as *IEX-1*, is an early response gene which is induced by a variety of stimuli, e.g., UV light, hormones, toxins and biomechanical stress [12]. Its role in regulating apoptosis is cell type and stimulus dependent and can act as a pro- or as an anti-apoptotic factor [13]. However, the exact cellular conditions that favor either the pro-apoptotic activity of IER3 or its potential to protect cells from apoptosis are barely understood [14].

In the cardiovascular system, studies have shown that mechanical overload leads to induction of *IER3* in cardiomyocytes, in which IER3 plays an anti-hypertrophic role [15]. In a Lamin A/C haploinsufficiency mouse model, activation of *IER3* expression was impaired under pressure overload [16].

The role of *IER3* in heart failure is barely understood. Here, we examined *IER3* in a murine cardiomyocyte tumor cell line (HL-1 cells) and in an inducible DCM mouse model harboring a human *TTN* truncation mutation. Understanding the pathophysiology of the disease might contribute to developing novel preventive or curative strategies for heart failure.

## 2. Results

### 2.1. Heterozygous Ttn Knock-In Mice Exhibit Enhanced Apoptosis during Dilated Cardiomyopathy Development

To investigate the role of apoptosis in *TTN*-based dilated cardiomyopathy, we used an inducible DCM mouse model with a human *TTN* truncation mutation [6]. We have shown previously that homozygous *Ttn* knock-in mice die in utero due to severe defects in sarcomeric assembly. However, the heterozygous littermates do not show an overt cardiac phenotype under resting conditions, but develop a DCM-like pattern under cardiac stress [7], therefore recapitulating the human phenotype.

We induced DCM in heterozygous *Ttn* knock-in mice by Ang II infusion as performed previously [7] and evaluated apoptosis levels by terminal deoxynucleotidyl transferase dUTP nick end labeling (TUNEL) and ssDNA stainings.

**Figure 1:**
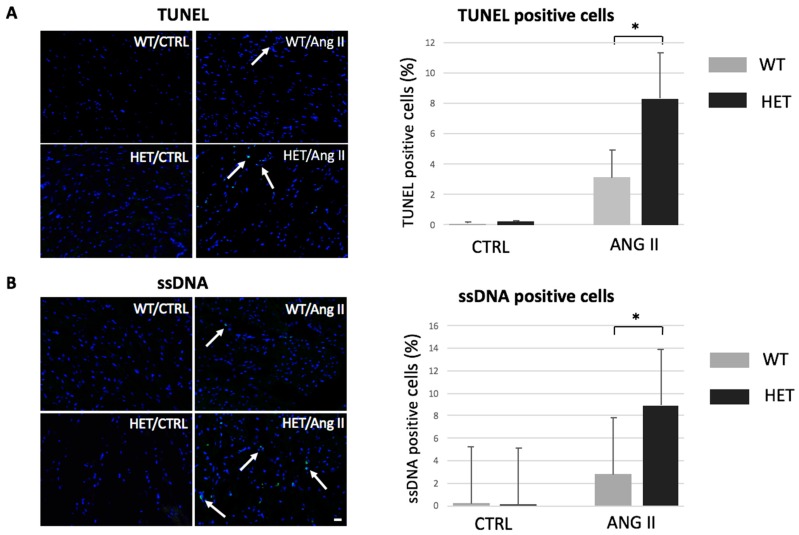
Enhanced apoptosis in titin-deficient animals developing heart failure. (A) Representative images and quantitative analysis of transferase dUTP nick end labeling (TUNEL) stainings of heart sections of wildtype (WT) and heterozygous (HET) *Ttn* knock-in mice subjected to Ang II infusion. Arrows indicate TUNEL-positive nuclei (green). The slides were counterstained with 4′,6-Diamidino-2-Phenylindole (DAPI, blue). The bar graph indicates percentage of TUNEL-positive cells (*n* = 3 for the control groups infused with saline, *n* = 6 for each group infused with Ang II, * *p* < 0.05); (B) Representative images and analysis of single-stranded DNA (ssDNA) staining on heart sections of mice subjected to Ang II infusion. Arrows indicate ssDNA-positive nuclei (green). Scale bar: 20 μm. The slides were counterstained with DAPI (blue). The bar graph indicates percentage of ssDNA-positive cells in the heart (*n* = 3 for the control groups infused with saline, *n* = 5 for each group infused with Ang II, * *p* < 0.05).

Under baseline conditions, apoptosis levels in wildtype (WT) and heterozygous (HET) animals were extremely low and barely detectable. Ang II infusion for 14 days at pharmacological doses (14 mg/kg/day) resulted in increased apoptosis in both groups, WT and HET mice. However, the HET animals exhibited significantly more TUNEL-positive cardiomyocytes compared to the WT mice (8.26% ± 3.6% vs. 3.18% ± 1.8%, *p* < 0.05, Figure 1A). Cardiac apoptosis was further examined by ssDNA stainings. In accordance with the TUNEL results, the HET mice showed increased levels of apoptosis compared to the WT mice (9.06% ± 0.4% vs. 2.48% ± 0.2%, *p* < 0.05, Figure 1B).

### 2.2. Heterozygous Ttn-Deficient Mice Show Impaired IER3 Signaling

*IER3* is a regulator of apoptosis in a variety of organs [14,17,18,19,20]. To investigate the role of *IER3* signaling in heart failure, we analyzed *IER3* expression during DCM development in heterozygous titin-deficient mice.

**Figure 2:**
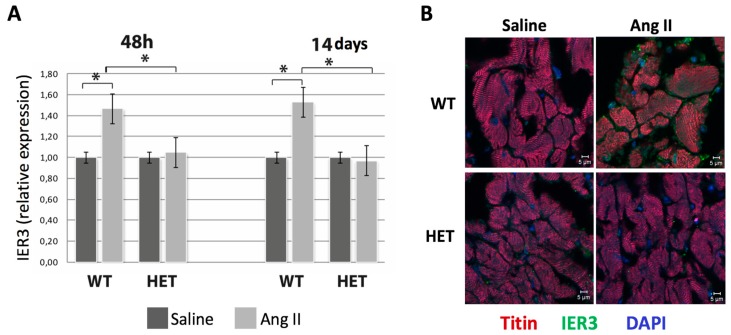
Impaired cardiac *IER3* activation in the titin-deficient mice during heart failure development. (**A**) Real-time semi-quantitative polymerase chain reaction (PCR) analysis of cardiac *IER3* expression in WT and HET *Ttn* knock-in mice 48 h and two weeks after Ang II infusion (*n* = 8, * *p* < 0.05). *IER3* response is blunted in the HET mice; (**B**) Representative IER3 immunofluorescence staining in WT and HET *Ttn* knock-in mice 48 h after Ang II infusion. Titin staining (red) was used to label the myocardium, nuclei were counterstained with DAPI (blue). Scale bar: 5 μm.

Real-time polymerase chain reaction (PCR) was performed 48 h after Ang II mini-pump implantation (before detectable onset of disease) and after two weeks (full DCM phenotype). Ang II infusion induced *IER3* expression in WT animals after 48 h (1.48 ± 0.51-fold, *p* < 0.05, Figure 2A) and after 14 days (1.55 ± 0.47-fold, *p* < 0.05, Figure 2A). Interestingly, the cardiac *IER3* response was blunted in the HET mice at 48 h (1.07 ± 0.85-fold, n.s.) and two weeks after Ang II infusion (0.93 ± 1.02-fold, n.s.). *IER3* expression levels were further assessed by IER3 immunofluorescence staining. In WT mice, Ang II infusion resulted in a slight expression of *IER3*, mainly in the cardiomyocyte nucleus and the peri-nuclear region, whereas IER3 signals were barely detectable in HET hearts (Figure 2B).

### 2.3. IER3 Is Involved in the Regulation of Apoptosis in Cardiomyocytes

In order to reveal *IER3*-dependent gene network regulation in the mouse heart and to assess its role in apoptosis, chromatin immunoprecipitation (ChIP) assays were performed using an anti-IER3 antibody. IER3 chromatin precipitates were separated and DNA fragments were analyzed by next-generation sequencing. The enriched sequences were clustered by bioinformatics methods (the full list of enriched sequences can be provided upon request).

We found that IER3 targets promotor regions of genes that are involved in apoptosis. However, we also detected enriched promotor sequences that are involved in metabolic, cellular, developmental and immune processes.

**Figure 3:**
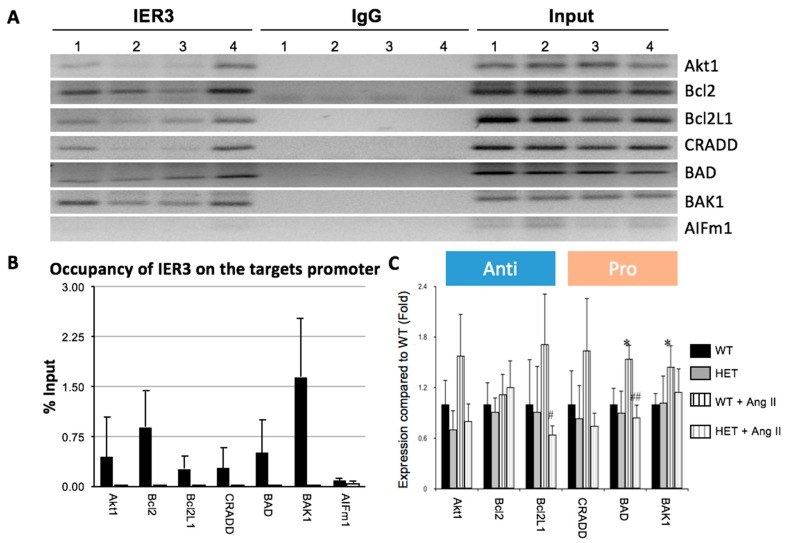
IER3 targets promotor regions of apoptosis genes in the murine heart. (**A**) Chromatin immunoprecipitation-PCR (ChIP-PCR) analysis of IER3 target promotor regions. IgG ChIP samples were included as negative control. *AIFm1* is a non-related negative control. Heart lysates from four different animals (1–4) were analyzed; (**B**) Percentages of IER3 occupation on its targets promoter regions. Percentage were calculated as the ratio of occupation of ChIP products to input (*p* < 0.05 for target promotor sites compared to control); (**C**) Real-time PCR analysis of IER3 target genes in mouse hearts infused with Ang II for two weeks. The expression of *BAD* and *BAK1* was significantly upregulated in the WT mice with Ang II infusion when compared to the WT with saline infusion, the expression of *Bcl2L1* and *BAD* in the HET mice with Ang II infusion was significantly reduced when compared to the WT mice with Ang II infusion (*n* = 4 for each group, * *p* < 0.05 compared to the wildtype mice infused with saline. # *p* < 0.05, ## *p* < 0.01 compared to wildtype mice infused with Ang II).

We confirmed the binding of IER3 to the promoters of apoptosis genes by PCR (Figure 3A). We found enrichment of promotor regions of the anti-apoptotic factors *Akt1*, *Bcl2*, and *Bcl2L1*, as well as of the pro-apoptotic factors Death Domain Containing Protein (*CRADD*), Bcl2-Associated Agonist of Cell Death (*BAD*) and Bcl2 Agonist/Killer 1 (*BAK1*) (Figure 3B).

We then investigated expression levels of these genes in *Ttn*-deficient mice after Ang II infusion for 14 days. Ang II treatment of WT mice resulted in an induction of anti-apoptotic genes *Akt1* (1.5-fold vs. baseline, *p* < 0.05) and *Bcl2L1* (1.75-fold vs. baseline, *p* < 0.05), while induction of these factors in HET mice was blunted (*Akt1*: 0.7-fold vs. baseline; *Bcl2L1*: 0.6-fold vs. baseline, n.s.). However, induction of the pro-apoptotic factors *CRADD*, *BAD* and *BAK* was also stronger in WT animals compared to HET littermates.

### 2.4. IER3 Is Anti-Apoptotic in HL-1 Cardiomyocytes

**Figure 4:**
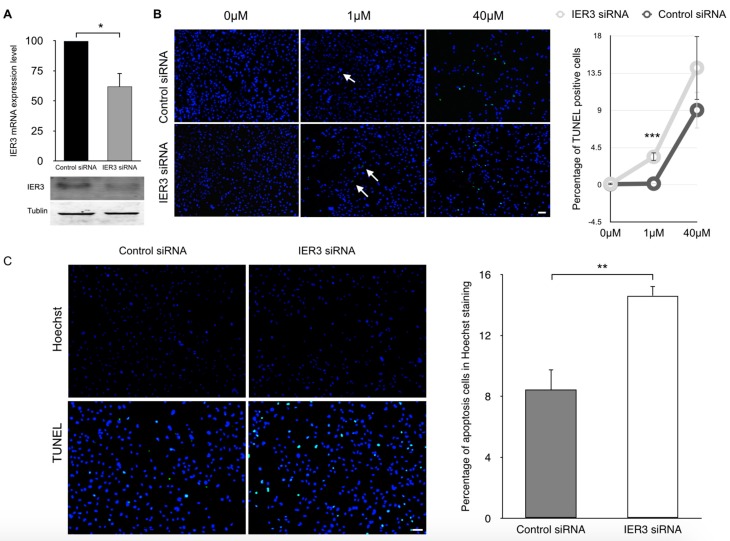
*IER3* knock-down sensitizes HL-1 cardiomyocytes to apoptosis. (**A**) Real-time PCR evaluation of *IER3* expression in HL-1 cardiomyocytes after knock-down. Glyceraldehyde-3-Phosphate Dehydrogenase (*GAPDH*) was used as an internal control (*n* = 3 for each group, * *p* < 0.05); (**B**) Representative TUNEL stainings of siRNAs transfected HL-1 cells treated with different concentrations of doxazosin. Arrows indicate TUNEL-positive nuclei (green). Scale bar: 50 μm. The bar graph indicates percentage of TUNEL-positive cells (*n* = 3 for each group, *** *p* < 0.001); (**C**) Representative images of Hoechst 3328 and TUNEL stainings of siRNAs-transfected HL-1 cells treated with heat shock (*n* = 3 for each group, ** *p* < 0.01). Scale bar: 50 μm.

To further elucidate the role of *IER3* in regulating apoptosis in cardiomyocytes, we performed RNAi mediated loss-of-function studies under various conditions that induce apoptosis. We used two models of apoptosis on HL-1 cells: heat shock and treatment with Doxazosin. HL-1 cells were transfected with a pool of siRNAs targeting *IER3* which could reduce *IER3* expression by 40% compared to control siRNAs (Figure 4A).

TUNEL and ssDNA stainings revealed that treatment with Doxazosin at a concentration of 1 μM and 40 μM induced apoptosis in HL-1 cells transfected with either siRNAs. However, induced apoptosis was stronger in *IER3*-silenced cells compared to control siRNA-transfected cells at a concentration of 1 μM Doxazosin; it was also stronger at a concentration of 40 μM Doxazosin, but the difference was not significant (3.4% vs. 0.1% for 1 μM Doxazosin, *p* < 0.001, Figure 4B).

In addition, *IER3*-silenced cells were subjected to heat shock. Heat shock treatment induced higher level of apoptosis in *IER3*-silenced cardiomyocytes compared to control siRNA-transfected cells (14.6% vs. 8.4%, *p* < 0.05, Figure 4C). These results indicate that the *IER3* knock-down sensitizes HL-1 cells to apoptosis, suggesting that *IER3* plays a predominantly anti-apoptotic role in cardiomyocytes under stress conditions.

## 3. Discussion

Apoptosis is an important mechanistic factor in heart failure as it induces cell loss and interrupts the myocardial integrity [21]. The early response protein IER3 is released upon a variety of cellular stress stimuli and regulates apoptosis in multiple cell types and conditions [12,13,14,15,17,18,19,20,22,23]. Depending on the cellular context, it can act as a pro- or as an anti-apoptotic factor.

Although *IER3* is expressed ubiquitously, a constitutive *IER3* knock-out mouse exhibits a cardiac phenotype with hypertension and cardiac hypertrophy [24], indicating that the function of this gene is important for the cardiovascular system. However, the exact mechanism remains unclear. In addition, upregulation of *IER3* was observed in myocardium and cardiomyocytes in response to mechanical stress [15]. However, the role of *IER3* in heart failure, and particularly in titin-based dilated cardiomyopathy, has not been investigated so far.

In accordance with other human and animal studies [9], we found that apoptosis is activated during heart failure development. We investigated *IER3* expression and demonstrated a blunted expression in *Ttn*-deficient animals. Chromatin-immunoprecipitation assays revealed that IER3 proteins can bind to the promotor of anti- and pro-apoptotic genes. However, knocking-down *IER3* in cardiomyocytes activated the apoptotic response of the cells upon various stimuli. Our data therefore indicate that *IER3* is a predominantly anti-apoptotic factor in cardiomyocytes in vitro. In addition, proper activation of *IER3* expression in the heart might be involved in cardiac resistance to adverse cardiac remodeling processes.

The regulation of the release of apoptotic factors is modulated by the *Bcl2* family members (e.g., *Bcl2*, *Bcl2L1*, *Bad*, *Bak*, and *Bax*) [25,26]. The protective role of anti-apoptotic *Bcl2* in the heart has been demonstrated by the fact that cardiac-specific overexpression of *Bcl2* significantly reduces infarct size after I/R [27]. In addition, *Bax* deletion reduces cardiac infarction size and improves cardiac function in mice after myocardial infarction [28]. Our study shows that IER3 targets anti-apoptotic factors *Bcl2*, *Bcl2L1* and *Akt1, and* acts as a suppressor of apoptosis in cardiomyocytes. Blunted *IER3* response may be an important mechanism for heart failure in our mouse model.

We can neither confirm nor exclude that dysregulated *IER3* expression is specific to a mutation in *Ttn*. We performed yeast-2-hybrid studies in order to demonstrate a physical interaction between titin and IER3, but we failed to prove this interaction. It is possible that titin and IER3 interact through a yet-undefined factor. Further studies are required to pinpoint exact pathways involved in *IER3* expression in the *Ttn* knock-in mouse model.

**Figure 5:**
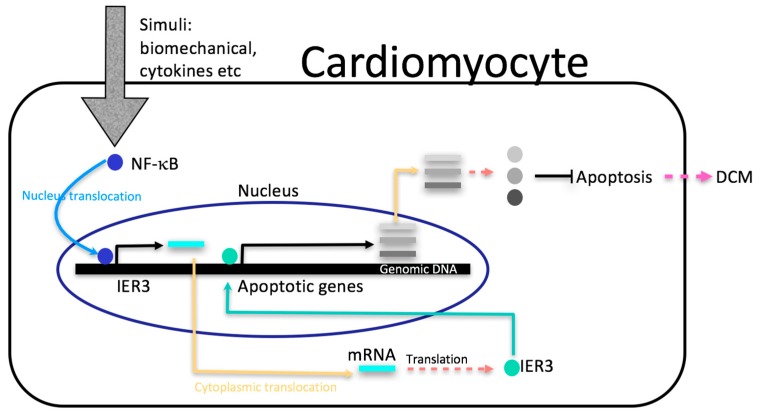
Illustration of the possible role of *IER3* in the regulation of cardiac apoptosis and dilated cardiomyopathy (DCM) development. Various stimuli activate *NF-κB* nucleus translocation (blue curved line). In turn, binding of *NF-κB* on the promoter of *IER3* positively regulates *IER3* transcription (black arrow turned right). After pre-mRNA processing, the *IER3* mature mRNAs are exported from the nucleus to the cytoplasm (yellow arrow turned right), where they are translated into IER3 proteins (red dashed arrow). Then, IER3 proteins translocate into the cell nuclei (green arrow turned left and up), which binds to the promoter of apoptotic genes and regulates their transcription (black arrow turned right). After pre-mRNA processing, the mRNA of apoptotic genes is exported to the cytoplasm (yellow arrow turned right), and translated to proteins (red dashed arrow). The apoptotic proteins play an anti-apoptotic role in cardiomyocytes (black blunt-end line). Cardiac apoptosis is involved in the development of dilated cardiomyopathy (pink dashed arrow).

Based on previous [14,17,22,29,30] and present work, we propose a model for the role of *IER3* in DCM development: *Gq*/*11*-coupled receptor agonists like Angiotensin II and other stimuli can activate canonical *NF-κB* signalling in the heart [31]. On the other hand, *IER3* is a direct target of *NF-κB* [22]. Our work has shown that *IER3* regulates promotors of apoptosis genes and acts as an anti-apoptotic factor in the heart. In our DCM mouse model, harbouring a titin truncation mutation, *IER3* signalling is impaired, which resulted in an enhanced apoptotic response. The titin truncation mutation affects its M-band region, which is a hotspot for sarcomeric protein–protein interaction and myofibrillar signalling and therefore is a good candidate to influence this pathway directly or indirectly [6]. The integration of a truncated titin protein could result in an impaired sarcomeric response to mechanical or neurohumoral challenges. Since apoptosis is an important mechanistic factor in heart failure as it induces cell loss and interrupts the myocardial integrity, this pathway might play a role in the pathological remodelling during DCM development (Figure 5).

Taken together, our results indicate that *IER3* plays an anti-apoptotic role in the heart and regulates the expression of a group of apoptosis genes. *Ttn*-deficient mice show an impaired activation of *IER3* under cardiac stress, which is associated with enhanced apoptosis. The inhibition of cardiac apoptosis through this pathway might be an effective therapeutic strategy to combat heart failure.

## 4. Materials and Methods

### 4.1. SiRNA Transfections in HL-1 Cells

HL-1 cardiomyocytes were kindly provided by William Claycomb (Louisiana State University Medical Center, New Orleans, LA, USA), and were cultured and maintained as described [32]. *IER3* and control siRNA (Dharmacon, Darmstadt, Germany) were used to silence *IER3* expression in HL-1 cells or served as negative control. One day before transfection, HL-1 cells were split and re-plated in six-well tissue culture dishes at a density of 5 × 10^5^ cells per well in antibiotic-free full-complement Claycomb medium (Sigma-Aldrich, St. Louis, MO, USA). The next day, siRNA (25 nM final concentration) was transfected with DharmaFECT 3 transfection reagent (Dharmacon) according to the manufacturer’s instructions; 24 h later, RNA was extracted and real-time PCR was performed to quantify the knock-down efficiency.

Twenty-four hours after siRNA transfection of HL-1 cells, the potent apoptosis inducer Doxazosin (Sigma-Aldrich) was added to the cell culture medium. Cells were maintained for 12 h in the cell culture incubator (37 °C, 5% CO_2_). Hoechst 33258 (Sigma-Aldrich) was added to the culture medium and the dishes were gently rocked and incubated for 45 min, after washing with Phosphate Buffered Saline (PBS) a cover glass was reversely mounted on a microscope slide. Six random areas per sample were acquired with a Zeiss microscopy (Zeiss, Jena, Germany) and analyzed with ImageJ (1.48 V, National Institutes of Health, Bethesda, MD, USA).

To confirm the role of *IER3* in HL-1 cardiomyocytes, we used a heat shock protocol to induce apoptosis. Twenty-four hours after siRNA transfections, cell culture dishes were placed in a humid box to prevent evaporation of the medium and the box was incubated at 45 °C for an hour.

### 4.2. Real-Time PCR

HL-1 cells were detached by trypsinization and collected by centrifugation. Total RNA was extracted from the cells with the RNeasy total RNA extraction kit (Qiagen, Hilden, Germany). The concentration and purity of RNA was determined by a Nanodrop spectrometer (Thermo Scientific, Dreieich, Germany). Single-stranded cDNA was synthesized from 1 μg of RNA using the Transcriptor first-strand cDNA synthesis kit (Roche Applied Science, Mannheim, Germany) with Oligo(dT)_18_ primers. *IER3* expression was quantified by real-time polymerase chain reaction, using qPCR Master Mix SYBR Green (Eurogentec, Cologne, Germany) on a LightCycler 480 (Roche Applied Science). Glyceraldehyde-3-Phosphate Dehydrogenase (*GAPDH*) was amplified in parallel reactions and served as an internal control for normalization in data analysis. Data was analyzed with LightCycler’s built-in software. Every pair of primers was tested for their specificity. The sequence of primers can be found in Table S1.

### 4.3. Animal Experiments

The *Ttn* knock-in mouse model was described previously [8]. Adult heterozygous *Ttn*-deficient mice (2–3 months old) and wild-type littermates were used. As described previously [8], DCM was induced by Angiotensin II (Sigma-Aldrich) infusion for 14 days via an osmotic mini-pump (Charles River Laboratories, Wilmington, MA, USA) delivering 14 mg/kg/day Ang II. Mice hearts were harvested after 7 and after 14 days, cryoembedded and mounted on glass slides. The tissue was fixed in 4% Paraformaldehyde (PFA) at room temperature for 10 min, followed by permeabilization for 10 min in 0.01% Triton X-100 PBS solution and blocked with 10% normal donkey serum. An anti-TTN antibody (Myomedix, Heidelberg, Germany) and an anti-IER3 antibody (sc-8457, Santa Cruz Biotechnology, Heidelberg, Germany) were diluted 1:100 and applied to the tissue and incubated at 4 °C overnight. The next day, the tissue was washed three times and incubated with an Alexa fluor 568 donkey anti-rabbit secondary antibody and an Alexa fluor 488 donkey anti-goat secondary antibody at a dilution of 1:300 (Invitrogen, Darmstadt, Germany) at room temperature for 1 h. The tissue was washed again and counter-stained with 4′,6-Diamidino-2-Phenylindole (DAPI), sealed in glass and images were acquired with a fluorescent microscope (Zeiss). The study was carried out according to the guidelines for the Care and Use of Laboratory Animals of the National Institutes of Health. The protocol M3/13 was approved on 28th April 2013 by the Committee on Ethics of Animal Experiments of the University Hospital Tuebingen.

### 4.4. Chromatin Immunoprecipitation

The chromatin immunoprecipitation (ChIP) procedure was performed at 4 °C. A corning dish was pre-cooled on ice. Hearts from adult mice were dissected, placed on the dish and chipped into 1–3 mm^3^ blocks. The heart pieces were transferred to a 15 mL Falcon tube (Corning, Wiesbaden, Germany) containing 2 mL of pre-cooled PBS with proteinase inhibitors (Roche Applied Science, Mannheim, Germany); 37% formaldehyde (Fischer Scientific, Schwerte, Germany) was added at a final concentration of 1.5% for fixation. The cross-linking reaction was terminated by adding 100 μL of 2.5 M Glycine (Carl Roth, Karlsruhe, Germany). Tissue was pelleted by centrifugation and was washed with pre-cooled PBS. The tissue pellet was lysed in 2 mL pre-cooled FA Lysis Buffer (which consists of 0.61 g of Tris (Sigma-Aldrich, St. Louis, MO, USA), 0.8 g NaCl (AppliChem, Darmstadt, Germany), 20 mL of 0.5 M EDTA (Invitrogen, Darmstadt, Germany) and 1 mL Triton X-100 (Sigma-Aldrich, St. Louis, MO, USA), 1 mL of 10% Sodium Deoxycholate (AppliChem, Darmstadt, Germany) and 0.1 g SDS (AppliChem, Darmstadt, Germany), 2 tablets proteinase inhibitor per 100 mL) on ice for 30 min. Then, chromatin was sheared by ultrasound sonication with a Sonifior 250 (Branson, Dietzenbach, Germany). The homogeneous lysate was centrifuged at 20,000× *g* for 5 min. An anti-IER3 antibody (ab65152, Abcam, Cambridge, UK) was coupled with Dynabeads protein A/G (MAGnify Chromatin Immunoprecipitation System, Invitrogen, Darmstadt, Germany). The antibody-Dynabeads complexes with a final concentration of 5 μg/μL were incubated with the heart lysate. Rabbit IgG (the MAGnify kit) was included as a negative control at the same concentration. The samples were incubated overnight with end-over-end rotation. The next day, the beads were pelleted with a magnet and washed with Buffer 1 and 2 (the MAGnify kit). DNA-antibody complexes were eluted by incubation with Washing Buffer (100 μL of 10% SDS, 100 μL of 1M NaHCO3 (AppliChem, Darmstadt, Germany) and 800 μL ddH_2_O), and DNA was released by subsequently serial incubation with 0.2 M NaCl, 0.036 M EDTA and 1 μL proteinase K (the MAGnify kit). DNA was purified with the Qiagen PCR purification kit (Qiagen, Hilden, Germany) and eluted in ddH_2_O for further analysis.

### 4.5. ChIP Sequencing and Analysis

ChIP sequencing and annotations were performed at the Microarray Facility Tuebingen Services at the Department of Human Genetics, University Hospital of Tuebingen. After sequencing, raw data was aligned with the mm9 mouse reference genome in bowtie. Model-based Analysis for ChIP-Seq (MACS) was used for peak calling with input DNA as a control. Peaks were filtered according to fold enrichment and False Discovery Rate (FDR). The genomic intervals of the triplicate data sets were concatenated and merged. The resulting intervals were intersected with the mouse genome, for which the 1000 bp upstream transcription start sites served as putative promoter region to find the genes as potential targets.

The potential IER3 binding motif was found by analyzing the ChIP sequencing results. Therefore, the tags from the ChIP datasets and mouse genomic promoter intervals were imported into Galaxy/Cistromes [33,34]. The resulting sequence was further processed by adding 80 bp to each flank. Multiple EM for Motif Elicitation (MEME) [35] was used to identify potential IER3 binding motifs. Minimal motif width from 6 to 4 and maximum motif width from 6–10, 14 and 50 were tested. The IER3 binding motif was confirmed by ChIPMotifs [36], Suite for Computational identification Of Promoter Elements (SCOPE) [37] and Discovering Ranked Imbalanced Motifs using suffix trees (DRIMust) [38] and then analyzed by Find Individual Motif Occurrences (FIMO) [39] for identification of potential IER3 targets. From the matches, two anti-apoptotic (*Akt1* and *Bcl2L1*) and three pro-apoptotic genes (*CRADD*, *BAD* and *BAK1*) were selected for ChIP-PCR analysis. Meanwhile, *Bcl2* and *Bcl2L1* were experimentally confirmed as IER3 targets by another study. The full list of enriched sequences can be found in Table S2.

### 4.6. TUNEL Stainings

Roche fluorescein TUNEL kit (Roche Applied Science, Mannheim, Germany) was used to assess apoptosis in mouse heart tissue and HL-1 cells. The TUNEL staining was performed according to the manufacturer’s instructions. Briefly, tissue on slides was fixed with 4% PFA for 10 min at room temperature. After rinsing with PBS, the slides were permeabilized with 0.1% Triton X-100/PBS for 10 min. TUNEL staining was carried out with incubation reaction solution (5 μL of enzyme solution mixed with 45 μL of labelling solution) for 1 h at 37 °C. The negative control (slide incubated with labelling solution only) and positive control (DNase I-treated slide incubated with reaction solution) were included to confirm the specificity of the stainings. Slides were washed with PBS, counterstained with DAPI and mounted.

We used three control mice for saline infusion and six mice for infusion with Ang II. For each mouse heart, six random areas from one slide per sample were photographed and analyzed with low magnification on a Zeiss fluorescent microscope. Analysis was performed with ImageJ.

### 4.7. Single-Stranded DNA Stainings

Single-stranded DNA (ssDNA) staining was applied on cryopreserved mouse heart samples. Heart tissue slides were fixed and permeabilized as described above, followed by a 50% Formamide/PBS bath at 95 °C for 10 min. After cooling down to room temperature, the slides were pre-incubated with 1% Bovine Serum Albumin (BSA)/PBS. An anti-ssDNA antibody (IBL, Fujioka, Japan) was diluted 1:20 in 1% BSA/PBS, applied to slides and incubated at 4 °C overnight. The next day, the slides were washed with PBS, incubated with an Alexa fluor 488 labelled anti-rabbit antibody (1:300 in 1% BSA/PBS) and counterstained with DAPI.

### 4.8. Statistics

Statistical analyses were performed with the 2-tailed Student’s *t*-test for the comparison of two groups. All data are shown as mean ± SD. A *p*-value ≤ 0.05 was considered statistically significant.

## Supplementary Materials

Supplementary materials can be found at www.mdpi.com/1422-0067/18/4/723/s1.
