# Supplementary material for: Dysregulated IER3 Expression is Associated with Enhanced Apoptosis in Titin-Based Dilated Cardiomyopathy
Source: Int J Mol Sci. 2017 Mar 29;18(4):723. doi: 10.3390/ijms18040723 (PMC5412309; doi:10.3390/ijms18040723)
Supplement: Supplementary file 1 [file ijms-18-00723-s001.pdf]

Zhou et al. Dysregulated IER3 expression is associated with enhanced apoptosis in titin-based dilated cardiomyopathy

Supplemental Table 1

List of primer sequences

Primer Name	Primer Sequence
IEX-1-RT-for	**CAGCCGAAGGGTGCTCTAC**
IEX-1-RT-rev	**AAATCTGGCAGAAGATGATGG**
Akt1-RT-for	**ATGAACGACGTAGCCATTGTG**
Akt1-RT-rev	**TTGTAGCCAATAAAGGTGCCAT**
Bcl-2-RT-for	**ATGCCTTTGTGGAACTATATGGC**
Bcl-2-RT-rev	**GGTATGCACCCAGAGTGATGC**
Bcl2l1-RT-for	**GACAAGGAGATGCAGGTATTGG**
Bcl2l1-RT-rev	**TCCCGTAGAGATCCACAAAAGT**
CRADD-RT-for	**AAGGCGAGAGAGGAAGTCACA**
CRADD-RT-rev	**GTTAATCTGCTGGTCTGATGGC**
BAD-RT-for	**GAGGAGGAGCTTAGCCCTTT**
BAD-RT-rev	**AGGAACCCTCAAACTCATCG**
BAK1-RT-for	**ATATTAACCGGCGCTACGAC**
BAK1-RT-rev	**AGGCGATCTTGGTGAAGAGT**
GAPDH-RT-for	**ATCTTCTTGTGCAGTGCCAGCCTCGTC**
GAPDH-RT-rev	**CTTCCCATTCTCGGCCTTGACTGTGC**
Akt1-ChIP-for	**CCCCCAAGCCTCACCCATCTG**
Akt1-ChIP-rev	**CCAACCCAGTACTGCCTGCCACAG**
Bcl-2-ChIP-for	**CAGCCGCAGCCGTCACCGTG**
Bcl-2-ChIP-rev	**GCTGCGCACCCCTTTCTCCTCCTC**
Bcl2l1-ChIP-for	**CAGCACGAGCAGTCAGCCAGGTAG**
Bcl2l1-ChIP-rev	**AGGCCCAGTGGCTTCCAGACAC**
CRADD-ChIP-for	**TCGTCGGTAGCTCTAACTGTGCCAC**
CRADD-ChIP-rev	**AGGTGAGGAGGGCGTGGGTGAC**
BAD-ChIP-for	**GGGCAGCGTACGCACACCTATC**
BAD-ChIP-rev	**TCGGGAGCTCCTTTACGGCAG**
BAK1-ChIP-for	**CCTCTATTTCATGATCCTCCTGCTTC**
BAK1-ChIP-rev	**CGGGCCTCCTCTTCCATCTTCTC**
AIFm1-ChIP-for	**GGTTTGAATGTGTCATGGCCACTGTC**
AIFm1-ChIP-rev	**CAGACAGGAGGGAGGCAAAGGACAG**

Zhou et al. Dysregulated IER3 expression is associated with enhanced apoptosis in titin-based dilated cardiomyopathy

Supplemental Table 2

IER3 ChiP-Seq: List of enriched sequences

FIMO analysis of IER3 target

#pattern name	sequence name	start	stop	strand	score	p-value	q-value	matched sequence
1	mm10_knownGene_uc008bhj.2	1	7	+	10.8143	9.74e-05	0.253	CTCCCTG
1	mm10_knownGene_uc008zvu.2	1	7	+	10.8143	9.74e-05	0.253	CTCCCTG
1	mm10_knownGene_uc029srq.1	1	7	+	10.8143	9.74e-05	0.253	CTTCCTG
1	mm10_knownGene_uc007ziu.1	1	7	+	10.8143	9.74e-05	0.253	CTTCCTG
1	mm10_knownGene_uc029ufu.1	1	7	+	10.8143	9.74e-05	0.253	CTTCCTG
1	mm10_knownGene_uc008rxa.1	1	7	+	10.8143	9.74e-05	0.253	CTTCCTG
1	mm10_knownGene_uc008zwd.2	1	7	+	10.8143	9.74e-05	0.253	CTCCCTG
1	mm10_knownGene_uc009fyh.1	1	7	+	10.8143	9.74e-05	0.253	CTTCCTG
1	mm10_knownGene_uc007aot.3	1	7	+	10.8143	9.74e-05	0.253	CTTCCTG
1	mm10_knownGene_uc008zvw.2	1	7	+	10.8143	9.74e-05	0.253	CTCCCTG
1	mm10_knownGene_uc012esy.1	1	7	+	10.8143	9.74e-05	0.253	CTTCCTG
1	mm10_knownGene_uc012esx.1	1	7	+	10.8143	9.74e-05	0.253	CTTCCTG
1	mm10_knownGene_uc029rfq.1	1	7	+	10.8143	9.74e-05	0.253	CTTCCTG
1	mm10_knownGene_uc008zvy.2	1	7	+	10.8143	9.74e-05	0.253	CTCCCTG
1	mm10_knownGene_uc056zpe.1	1	7	+	10.8143	9.74e-05	0.253	CTTCCTG
1	mm10_knownGene_uc008zvz.2	1	7	+	10.8143	9.74e-05	0.253	CTCCCTG
1	mm10_knownGene_uc008zwe.2	1	7	+	10.8143	9.74e-05	0.253	CTCCCTG
1	mm10_knownGene_uc008zwc.2	1	7	+	10.8143	9.74e-05	0.253	CTCCCTG
1	mm10_knownGene_uc012bbl.1	1	7	+	10.8143	9.74e-05	0.253	CTTCCTG
1	mm10_knownGene_uc012dah.1	1	7	+	10.8143	9.74e-05	0.253	CTTCCTG
1	mm10_knownGene_uc009dsh.1	1	7	+	10.8143	9.74e-05	0.253	CTCCCTG
1	mm10_knownGene_uc009cmd.1	1	7	+	10.8143	9.74e-05	0.253	CTCCCTG
1	mm10_knownGene_uc008gms.1	1	7	+	10.8143	9.74e-05	0.253	CTCCCTG
1	mm10_knownGene_uc008zwh.2	1	7	+	10.8143	9.74e-05	0.253	CTCCCTG

1	mm10_knownGene_uc009dsg.1	1	7	+	10.8143	9.74e-05	0.253	CTCCCTG
1	mm10_knownGene_uc008zvx.2	1	7	+	10.8143	9.74e-05	0.253	CTCCCTG
1	mm10_knownGene_uc008zvv.2	1	7	+	10.8143	9.74e-05	0.253	CTCCCTG
1	mm10_knownGene_uc008zwa.2	1	7	+	10.8143	9.74e-05	0.253	CTCCCTG
1	mm10_knownGene_uc008zwb.2	1	7	+	10.8143	9.74e-05	0.253	CTCCCTG
1	mm10_knownGene_uc009fyk.1	1	7	+	10.8143	9.74e-05	0.253	CTTCCTG
1	mm10_knownGene_uc057bgi.1	2	8	+	10.8143	9.74e-05	0.253	CTTCCTG
1	mm10_knownGene_uc007ziy.3	2	8	+	10.8143	9.74e-05	0.253	CTTCCTG
1	mm10_knownGene_uc008kut.2	2	8	+	10.8143	9.74e-05	0.253	CTTCCTG
1	mm10_knownGene_uc008yhe.1	2	8	+	10.8143	9.74e-05	0.253	CTTCCTG
1	mm10_knownGene_uc009pky.1	2	8	+	10.8143	9.74e-05	0.253	CTTCCTG
1	mm10_knownGene_uc008cux.1	2	8	+	10.8143	9.74e-05	0.253	CTCCCTG
1	mm10_knownGene_uc009grd.2	2	8	+	10.8143	9.74e-05	0.253	CTCCCTG
1	mm10_knownGene_uc012bzh.1	2	8	+	10.8143	9.74e-05	0.253	CTTCCTG
1	mm10_knownGene_uc007zix.3	2	8	+	10.8143	9.74e-05	0.253	CTTCCTG
1	mm10_knownGene_uc008gij.1	2	8	+	10.8143	9.74e-05	0.253	CTCCCTG
1	mm10_knownGene_uc008vgj.2	2	8	+	10.8143	9.74e-05	0.253	CTTCCTG
1	mm10_knownGene_uc009pkz.1	2	8	+	10.8143	9.74e-05	0.253	CTTCCTG
1	mm10_knownGene_uc008vgl.2	2	8	+	10.8143	9.74e-05	0.253	CTTCCTG
1	mm10_knownGene_uc007tav.1	2	8	+	10.8143	9.74e-05	0.253	CTCCCTG
1	mm10_knownGene_uc009pla.1	2	8	+	10.8143	9.74e-05	0.253	CTTCCTG
1	mm10_knownGene_uc009siw.1	2	8	+	10.8143	9.74e-05	0.253	CTCCCTG
1	mm10_knownGene_uc007khj.2	2	8	+	10.8143	9.74e-05	0.253	CTCCCTG
1	mm10_knownGene_uc029sdv.1	2	8	+	10.8143	9.74e-05	0.253	CTCCCTG
1	mm10_knownGene_uc029vel.1	2	8	+	10.8143	9.74e-05	0.253	CTCCCTG
1	mm10_knownGene_uc009pxv.1	2	8	+	10.8143	9.74e-05	0.253	CTCCCTG
1	mm10_knownGene_uc008kuu.2	2	8	+	10.8143	9.74e-05	0.253	CTTCCTG
1	mm10_knownGene_uc008kva.2	2	8	+	10.8143	9.74e-05	0.253	CTTCCTG
1	mm10_knownGene_uc007ziz.2	2	8	+	10.8143	9.74e-05	0.253	CTTCCTG
1	mm10_knownGene_uc007khi.2	2	8	+	10.8143	9.74e-05	0.253	CTCCCTG
1	mm10_knownGene_uc008vgk.2	2	8	+	10.8143	9.74e-05	0.253	CTTCCTG

1	mm10_knownGene_uc007khh.2	2	8	+	10.8143	9.74e-05	0.253	CTCCCTG
1	mm10_knownGene_uc009fpw.1	2	8	+	10.8143	9.74e-05	0.253	CTTCCTG
1	mm10_knownGene_uc008sky.1	2	8	+	10.8143	9.74e-05	0.253	CTCCCTG
1	mm10_knownGene_uc029vnf.1	2	8	+	10.8143	9.74e-05	0.253	CTCCCTG
1	mm10_knownGene_uc009szy.1	2	8	+	10.8143	9.74e-05	0.253	CTCCCTG
1	mm10_knownGene_uc057aha.1	2	8	+	10.8143	9.74e-05	0.253	CTCCCTG
1	mm10_knownGene_uc008smo.1	2	8	+	10.8143	9.74e-05	0.253	CTCCCTG
1	mm10_knownGene_uc008rko.2	2	8	+	10.8143	9.74e-05	0.253	CTCCCTG
1	mm10_knownGene_uc009tfi.1	3	9	+	10.8143	9.74e-05	0.253	CTCCCTG
1	mm10_knownGene_uc008ldx.2	3	9	+	10.8143	9.74e-05	0.253	CTCCCTG
1	mm10_knownGene_uc009aiz.1	3	9	+	10.8143	9.74e-05	0.253	CTTCCTG
1	mm10_knownGene_uc008xvf.2	3	9	+	10.8143	9.74e-05	0.253	CTTCCTG
1	mm10_knownGene_uc007cpf.2	3	9	+	10.8143	9.74e-05	0.253	CTCCCTG
1	mm10_knownGene_uc008hbm.1	3	9	+	10.8143	9.74e-05	0.253	CTTCCTG
1	mm10_knownGene_uc029wgo.1	3	9	+	10.8143	9.74e-05	0.253	CTCCCTG
1	mm10_knownGene_uc029tfn.1	3	9	+	10.8143	9.74e-05	0.253	CTCCCTG
1	mm10_knownGene_uc029vfm.1	3	9	+	10.8143	9.74e-05	0.253	CTCCCTG
1	mm10_knownGene_uc007qpc.1	3	9	+	10.8143	9.74e-05	0.253	CTCCCTG
1	mm10_knownGene_uc007cpg.2	3	9	+	10.8143	9.74e-05	0.253	CTCCCTG
1	mm10_knownGene_uc008sma.2	3	9	+	10.8143	9.74e-05	0.253	CTCCCTG
1	mm10_knownGene_uc012dsf.1	3	9	+	10.8143	9.74e-05	0.253	CTTCCTG
1	mm10_knownGene_uc033geh.1	3	9	+	10.8143	9.74e-05	0.253	CTCCCTG
1	mm10_knownGene_uc007cpe.2	3	9	+	10.8143	9.74e-05	0.253	CTCCCTG
1	mm10_knownGene_uc012ado.1	3	9	+	10.8143	9.74e-05	0.253	CTTCCTG
1	mm10_knownGene_uc008gip.1	3	9	+	10.8143	9.74e-05	0.253	CTTCCTG
1	mm10_knownGene_uc008wlt.1	3	9	+	10.8143	9.74e-05	0.253	CTCCCTG
1	mm10_knownGene_uc033geg.1	3	9	+	10.8143	9.74e-05	0.253	CTCCCTG
1	mm10_knownGene_uc009qmk.2	3	9	+	10.8143	9.74e-05	0.253	CTTCCTG
1	mm10_knownGene_uc008wlp.1	3	9	+	10.8143	9.74e-05	0.253	CTCCCTG
1	mm10_knownGene_uc008unu.1	3	9	+	10.8143	9.74e-05	0.253	CTTCCTG
1	mm10_knownGene_uc007oib.4	3	9	+	10.8143	9.74e-05	0.253	CTCCCTG

1	mm10_knownGene_uc008sif.1	3	9	+	10.8143	9.74e-05	0.253	CTTCCTG
1	mm10_knownGene_uc009azs.1	4	10	+	10.8143	9.74e-05	0.253	CTTCCTG
1	mm10_knownGene_uc009azt.1	4	10	+	10.8143	9.74e-05	0.253	CTTCCTG
1	mm10_knownGene_uc009aqs.2	4	10	+	10.8143	9.74e-05	0.253	CTTCCTG
1	mm10_knownGene_uc007flq.2	4	10	+	10.8143	9.74e-05	0.253	CTTCCTG
1	mm10_knownGene_uc011xfn.2	4	10	+	10.8143	9.74e-05	0.253	CTTCCTG
1	mm10_knownGene_uc009pqn.1	4	10	+	10.8143	9.74e-05	0.253	CTCCCTG
1	mm10_knownGene_uc009gsd.1	4	10	+	10.8143	9.74e-05	0.253	CTCCCTG
1	mm10_knownGene_uc007wcg.2	4	10	+	10.8143	9.74e-05	0.253	CTCCCTG
1	mm10_knownGene_uc009duy.1	4	10	+	10.8143	9.74e-05	0.253	CTCCCTG
1	mm10_knownGene_uc029smx.1	4	10	+	10.8143	9.74e-05	0.253	CTTCCTG
1	mm10_knownGene_uc033hen.1	5	11	+	10.8143	9.74e-05	0.253	CTTCCTG
1	mm10_knownGene_uc008bdb.1	5	11	+	10.8143	9.74e-05	0.253	CTTCCTG
1	mm10_knownGene_uc008kjq.1	5	11	+	10.8143	9.74e-05	0.253	CTTCCTG
1	mm10_knownGene_uc009hmz.2	5	11	+	10.8143	9.74e-05	0.253	CTTCCTG
1	mm10_knownGene_uc007nxr.1	5	11	+	10.8143	9.74e-05	0.253	CTTCCTG
1	mm10_knownGene_uc029rco.1	5	11	+	10.8143	9.74e-05	0.253	CTCCCTG
1	mm10_knownGene_uc009mbr.2	6	12	+	10.8143	9.74e-05	0.253	CTCCCTG
1	mm10_knownGene_uc007gtm.1	6	12	+	10.8143	9.74e-05	0.253	CTTCCTG
1	mm10_knownGene_uc008fqj.1	6	12	+	10.8143	9.74e-05	0.253	CTCCCTG
1	mm10_knownGene_uc008auo.1	6	12	+	10.8143	9.74e-05	0.253	CTCCCTG
1	mm10_knownGene_uc007wtc.1	6	12	+	10.8143	9.74e-05	0.253	CTCCCTG
1	mm10_knownGene_uc009fse.1	6	12	+	10.8143	9.74e-05	0.253	CTCCCTG
1	mm10_knownGene_uc008aum.1	6	12	+	10.8143	9.74e-05	0.253	CTCCCTG
1	mm10_knownGene_uc009bio.2	6	12	+	10.8143	9.74e-05	0.253	CTCCCTG
1	mm10_knownGene_uc012czm.1	6	12	+	10.8143	9.74e-05	0.253	CTCCCTG
1	mm10_knownGene_uc011wpg.1	6	12	+	10.8143	9.74e-05	0.253	CTTCCTG
1	mm10_knownGene_uc012glc.1	6	12	+	10.8143	9.74e-05	0.253	CTCCCTG
1	mm10_knownGene_uc009nnq.1	6	12	+	10.8143	9.74e-05	0.253	CTCCCTG
1	mm10_knownGene_uc009iwd.1	6	12	+	10.8143	9.74e-05	0.253	CTTCCTG
1	mm10_knownGene_uc009bpr.4	6	12	+	10.8143	9.74e-05	0.253	CTCCCTG

1	mm10_knownGene_uc007wtb.1	6	12	+	10.8143	9.74e-05	0.253	CTCCCTG
1	mm10_knownGene_uc008bfi.2	6	12	+	10.8143	9.74e-05	0.253	CTTCCTG
1	mm10_knownGene_uc057car.1	6	12	+	10.8143	9.74e-05	0.253	CTTCCTG
1	mm10_knownGene_uc012gfi.1	6	12	+	10.8143	9.74e-05	0.253	CTCCCTG
1	mm10_knownGene_uc056yvx.1	6	12	+	10.8143	9.74e-05	0.253	CTTCCTG
1	mm10_knownGene_uc033foa.1	6	12	+	10.8143	9.74e-05	0.253	CTTCCTG
1	mm10_knownGene_uc007cal.1	6	12	+	10.8143	9.74e-05	0.253	CTTCCTG
1	mm10_knownGene_uc008lat.1	6	12	+	10.8143	9.74e-05	0.253	CTTCCTG
1	mm10_knownGene_uc007gzl.1	6	12	+	10.8143	9.74e-05	0.253	CTTCCTG
1	mm10_knownGene_uc007cak.1	6	12	+	10.8143	9.74e-05	0.253	CTTCCTG
1	mm10_knownGene_uc009nnp.1	6	12	+	10.8143	9.74e-05	0.253	CTCCCTG
1	mm10_knownGene_uc029xmo.1	6	12	+	10.8143	9.74e-05	0.253	CTTCCTG
1	mm10_knownGene_uc012ebj.1	6	12	+	10.8143	9.74e-05	0.253	CTTCCTG
1	mm10_knownGene_uc007cam.1	6	12	+	10.8143	9.74e-05	0.253	CTTCCTG
1	mm10_knownGene_uc029tlq.1	6	12	+	10.8143	9.74e-05	0.253	CTTCCTG
1	mm10_knownGene_uc009ntu.1	6	12	+	10.8143	9.74e-05	0.253	CTTCCTG
1	mm10_knownGene_uc007gtl.1	6	12	+	10.8143	9.74e-05	0.253	CTTCCTG
1	mm10_knownGene_uc033hpj.1	6	12	+	10.8143	9.74e-05	0.253	CTTCCTG
1	mm10_knownGene_uc057caq.1	6	12	+	10.8143	9.74e-05	0.253	CTTCCTG
1	mm10_knownGene_uc007hin.1	1	7	-	10.8143	9.74e-05	0.253	CTTCCTG
1	mm10_knownGene_uc007qkx.2	1	7	-	10.8143	9.74e-05	0.253	CTTCCTG
1	mm10_knownGene_uc007qrb.2	7	13	+	10.8143	9.74e-05	0.253	CTCCCTG
1	mm10_knownGene_uc007mra.1	1	7	-	10.8143	9.74e-05	0.253	CTTCCTG
1	mm10_knownGene_uc008sgb.2	7	13	+	10.8143	9.74e-05	0.253	CTCCCTG
1	mm10_knownGene_uc007hml.1	7	13	+	10.8143	9.74e-05	0.253	CTCCCTG
1	mm10_knownGene_uc008pgo.2	7	13	+	10.8143	9.74e-05	0.253	CTCCCTG
1	mm10_knownGene_uc029wqt.1	1	7	-	10.8143	9.74e-05	0.253	CTTCCTG
1	mm10_knownGene_uc009awb.1	1	7	-	10.8143	9.74e-05	0.253	CTTCCTG
1	mm10_knownGene_uc029uzl.1	1	7	-	10.8143	9.74e-05	0.253	CTTCCTG
1	mm10_knownGene_uc029ubx.1	1	7	-	10.8143	9.74e-05	0.253	CTCCCTG
1	mm10_knownGene_uc029xcr.1	1	7	-	10.8143	9.74e-05	0.253	CTTCCTG

1	mm10_knownGene_uc007hio.1	1	7	-	10.8143	9.74e-05	0.253	CTTCCTG
1	mm10_knownGene_uc009cld.1	1	7	-	10.8143	9.74e-05	0.253	CTTCCTG
1	mm10_knownGene_uc012dbn.1	7	13	+	10.8143	9.74e-05	0.253	CTCCCTG
1	mm10_knownGene_uc033jdn.1	1	7	-	10.8143	9.74e-05	0.253	CTTCCTG
1	mm10_knownGene_uc008pgn.2	7	13	+	10.8143	9.74e-05	0.253	CTCCCTG
1	mm10_knownGene_uc029wdw.1	7	13	+	10.8143	9.74e-05	0.253	CTTCCTG
1	mm10_knownGene_uc029rzp.1	1	7	-	10.8143	9.74e-05	0.253	CTTCCTG
1	mm10_knownGene_uc008img.1	1	7	-	10.8143	9.74e-05	0.253	CTTCCTG
1	mm10_knownGene_uc008oxy.1	7	13	+	10.8143	9.74e-05	0.253	CTTCCTG
1	mm10_knownGene_uc007cre.1	1	7	-	10.8143	9.74e-05	0.253	CTCCCTG
1	mm10_knownGene_uc007ihp.1	1	7	-	10.8143	9.74e-05	0.253	CTTCCTG
1	mm10_knownGene_uc009laz.1	1	7	-	10.8143	9.74e-05	0.253	CTTCCTG
1	mm10_knownGene_uc008pgr.2	7	13	+	10.8143	9.74e-05	0.253	CTCCCTG
1	mm10_knownGene_uc007hil.1	1	7	-	10.8143	9.74e-05	0.253	CTTCCTG
1	mm10_knownGene_uc008pgq.2	7	13	+	10.8143	9.74e-05	0.253	CTCCCTG
1	mm10_knownGene_uc007usr.1	1	7	-	10.8143	9.74e-05	0.253	CTTCCTG
1	mm10_knownGene_uc012cpr.1	7	13	+	10.8143	9.74e-05	0.253	CTCCCTG
1	mm10_knownGene_uc007nue.1	7	13	+	10.8143	9.74e-05	0.253	CTTCCTG
1	mm10_knownGene_uc008nqr.2	1	7	-	10.8143	9.74e-05	0.253	CTTCCTG
1	mm10_knownGene_uc009clc.1	1	7	-	10.8143	9.74e-05	0.253	CTTCCTG
1	mm10_knownGene_uc011xio.1	1	7	-	10.8143	9.74e-05	0.253	CTCCCTG
1	mm10_knownGene_uc029xnr.1	7	13	+	10.8143	9.74e-05	0.253	CTTCCTG
1	mm10_knownGene_uc008yyf.2	1	7	-	10.8143	9.74e-05	0.253	CTCCCTG
1	mm10_knownGene_uc029wrl.1	1	7	-	10.8143	9.74e-05	0.253	CTTCCTG
1	mm10_knownGene_uc029sai.1	1	7	-	10.8143	9.74e-05	0.253	CTCCCTG
1	mm10_knownGene_uc007hmm.1	7	13	+	10.8143	9.74e-05	0.253	CTCCCTG
1	mm10_knownGene_uc029tam.1	1	7	-	10.8143	9.74e-05	0.253	CTTCCTG
1	mm10_knownGene_uc029tdm.1	1	7	-	10.8143	9.74e-05	0.253	CTCCCTG
1	mm10_knownGene_uc009thv.2	1	7	-	10.8143	9.74e-05	0.253	CTTCCTG
1	mm10_knownGene_uc029ucl.1	1	7	-	10.8143	9.74e-05	0.253	CTCCCTG
1	mm10_knownGene_uc007chn.2	1	7	-	10.8143	9.74e-05	0.253	CTCCCTG

1	mm10_knownGene_uc009cle.1	1	7	-	10.8143	9.74e-05	0.253	CTTCCTG
1	mm10_knownGene_uc007gcj.2	1	7	-	10.8143	9.74e-05	0.253	CTCCCTG
1	mm10_knownGene_uc007him.1	1	7	-	10.8143	9.74e-05	0.253	CTTCCTG
1	mm10_knownGene_uc008pgp.2	7	13	+	10.8143	9.74e-05	0.253	CTCCCTG
1	mm10_knownGene_uc009gpk.1	7	13	+	10.8143	9.74e-05	0.253	CTTCCTG
1	mm10_knownGene_uc012cpq.1	7	13	+	10.8143	9.74e-05	0.253	CTCCCTG
1	mm10_knownGene_uc008ryt.1	1	7	-	10.8143	9.74e-05	0.253	CTCCCTG
1	mm10_knownGene_uc008iut.1	2	8	-	10.8143	9.74e-05	0.253	CTTCCTG
1	mm10_knownGene_uc008wnp.2	8	14	+	10.8143	9.74e-05	0.253	CTCCCTG
1	mm10_knownGene_uc029tfv.1	8	14	+	10.8143	9.74e-05	0.253	CTCCCTG
1	mm10_knownGene_uc012akw.1	2	8	-	10.8143	9.74e-05	0.253	CTCCCTG
1	mm10_knownGene_uc029vuy.2	8	14	+	10.8143	9.74e-05	0.253	CTCCCTG
1	mm10_knownGene_uc029uca.1	2	8	-	10.8143	9.74e-05	0.253	CTCCCTG
1	mm10_knownGene_uc012djj.1	2	8	-	10.8143	9.74e-05	0.253	CTTCCTG
1	mm10_knownGene_uc008xvz.2	8	14	+	10.8143	9.74e-05	0.253	CTCCCTG
1	mm10_knownGene_uc008bza.2	8	14	+	10.8143	9.74e-05	0.253	CTTCCTG
1	mm10_knownGene_uc029txy.1	8	14	+	10.8143	9.74e-05	0.253	CTCCCTG
1	mm10_knownGene_uc029qxw.1	8	14	+	10.8143	9.74e-05	0.253	CTTCCTG
1	mm10_knownGene_uc007emg.1	2	8	-	10.8143	9.74e-05	0.253	CTTCCTG
1	mm10_knownGene_uc008wnq.2	8	14	+	10.8143	9.74e-05	0.253	CTCCCTG
1	mm10_knownGene_uc009frb.1	2	8	-	10.8143	9.74e-05	0.253	CTTCCTG
1	mm10_knownGene_uc009tbi.1	8	14	+	10.8143	9.74e-05	0.253	CTTCCTG
1	mm10_knownGene_uc008hln.1	8	14	+	10.8143	9.74e-05	0.253	CTTCCTG
1	mm10_knownGene_uc007xfc.1	2	8	-	10.8143	9.74e-05	0.253	CTTCCTG
1	mm10_knownGene_uc008aou.2	2	8	-	10.8143	9.74e-05	0.253	CTCCCTG
1	mm10_knownGene_uc011zxr.1	2	8	-	10.8143	9.74e-05	0.253	CTTCCTG
1	mm10_knownGene_uc007bnq.1	8	14	+	10.8143	9.74e-05	0.253	CTTCCTG
1	mm10_knownGene_uc012akx.1	2	8	-	10.8143	9.74e-05	0.253	CTCCCTG
1	mm10_knownGene_uc009cdk.1	8	14	+	10.8143	9.74e-05	0.253	CTCCCTG
1	mm10_knownGene_uc007xfd.1	2	8	-	10.8143	9.74e-05	0.253	CTTCCTG
1	mm10_knownGene_uc011znp.1	8	14	+	10.8143	9.74e-05	0.253	CTTCCTG

1	mm10_knownGene_uc009uoi.1	2	8	-	10.8143	9.74e-
05	0.253	CTTCCTG				
1	mm10_knownGene_uc008aot.2	2	8	-	10.8143	9.74e-
05	0.253	CTCCCTG				
1	mm10_knownGene_uc007vds.1	3	9	-	10.8143	9.74e-
05	0.253	CTTCCTG				
1	mm10_knownGene_uc009tsy.2	3	9	-	10.8143	9.74e-
05	0.253	CTTCCTG				
1	mm10_knownGene_uc008gtq.1	3	9	-	10.8143	9.74e-
05	0.253	CTCCCTG				
1	mm10_knownGene_uc009muj.1	3	9	-	10.8143	9.74e-
05	0.253	CTCCCTG				
1	mm10_knownGene_uc009lax.3	3	9	-	10.8143	9.74e-
05	0.253	CTTCCTG				
1	mm10_knownGene_uc029yhl.1	3	9	-	10.8143	9.74e-
05	0.253	CTTCCTG				
1	mm10_knownGene_uc009aeh.2	9	15	+	10.8143	9.74e-
05	0.253	CTCCCTG				
1	mm10_knownGene_uc033gkk.1	3	9	-	10.8143	9.74e-
05	0.253	CTCCCTG				
1	mm10_knownGene_uc012cbl.1	3	9	-	10.8143	9.74e-
05	0.253	CTTCCTG				
1	mm10_knownGene_uc008lqf.1	9	15	+	10.8143	9.74e-
05	0.253	CTTCCTG				
1	mm10_knownGene_uc009mpa.2	3	9	-	10.8143	9.74e-
05	0.253	CTCCCTG				
1	mm10_knownGene_uc008ull.2	9	15	+	10.8143	9.74e-
05	0.253	CTTCCTG				
1	mm10_knownGene_uc007hts.2	9	15	+	10.8143	9.74e-
05	0.253	CTCCCTG				
1	mm10_knownGene_uc007wfr.2	9	15	+	10.8143	9.74e-
05	0.253	CTCCCTG				
1	mm10_knownGene_uc033gzq.1	3	9	-	10.8143	9.74e-
05	0.253	CTTCCTG				
1	mm10_knownGene_uc011yxf.1	3	9	-	10.8143	9.74e-
05	0.253	CTCCCTG				
1	mm10_knownGene_uc009iwk.3	9	15	+	10.8143	9.74e-
05	0.253	CTCCCTG				
1	mm10_knownGene_uc029xlp.1	3	9	-	10.8143	9.74e-
05	0.253	CTTCCTG				
1	mm10_knownGene_uc009iwj.3	9	15	+	10.8143	9.74e-
05	0.253	CTCCCTG				
1	mm10_knownGene_uc009rmb.1	3	9	-	10.8143	9.74e-
05	0.253	CTTCCTG				
1	mm10_knownGene_uc007ptu.1	3	9	-	10.8143	9.74e-
05	0.253	CTCCCTG				
1	mm10_knownGene_uc009htt.1	3	9	-	10.8143	9.74e-
05	0.253	CTTCCTG				
1	mm10_knownGene_uc012gho.1	3	9	-	10.8143	9.74e-
05	0.253	CTCCCTG				
1	mm10_knownGene_uc033fth.1	3	9	-	10.8143	9.74e-
05	0.253	CTCCCTG				
1	mm10_knownGene_uc029tmz.1	9	15	+	10.8143	9.74e-
05	0.253	CTTCCTG				
1	mm10_knownGene_uc007eos.2	9	15	+	10.8143	9.74e-
05	0.253	CTTCCTG				
1	mm10_knownGene_uc007cej.1	3	9	-	10.8143	9.74e-
05	0.253	CTTCCTG				
1	mm10_knownGene_uc029tqf.3	9	15	+	10.8143	9.74e-
05	0.253	CTCCCTG				
1	mm10_knownGene_uc008lsn.1	3	9	-	10.8143	9.74e-
05	0.253	CTTCCTG				

1	mm10_knownGene_uc007weq.2	9	15	+	10.8143	9.74e-05	0.253	CTTCCTG
1	mm10_knownGene_uc012ghe.1	9	15	+	10.8143	9.74e-05	0.253	CTTCCTG
1	mm10_knownGene_uc009eqo.1	10	16	+	10.8143	9.74e-05	0.253	CTTCCTG
1	mm10_knownGene_uc007rmc.2	10	16	+	10.8143	9.74e-05	0.253	CTCCCTG
1	mm10_knownGene_uc007pvh.1	4	10	-	10.8143	9.74e-05	0.253	CTTCCTG
1	mm10_knownGene_uc008pqz.1	4	10	-	10.8143	9.74e-05	0.253	CTTCCTG
1	mm10_knownGene_uc029qyc.1	4	10	-	10.8143	9.74e-05	0.253	CTCCCTG
1	mm10_knownGene_uc009rmn.1	10	16	+	10.8143	9.74e-05	0.253	CTCCCTG
1	mm10_knownGene_uc008yyv.1	10	16	+	10.8143	9.74e-05	0.253	CTTCCTG
1	mm10_knownGene_uc007rtd.2	4	10	-	10.8143	9.74e-05	0.253	CTTCCTG
1	mm10_knownGene_uc008iov.2	4	10	-	10.8143	9.74e-05	0.253	CTCCCTG
1	mm10_knownGene_uc008fsy.2	4	10	-	10.8143	9.74e-05	0.253	CTTCCTG
1	mm10_knownGene_uc009pow.2	10	16	+	10.8143	9.74e-05	0.253	CTTCCTG
1	mm10_knownGene_uc008lep.2	4	10	-	10.8143	9.74e-05	0.253	CTCCCTG
1	mm10_knownGene_uc008ksv.2	10	16	+	10.8143	9.74e-05	0.253	CTTCCTG
1	mm10_knownGene_uc057bdl.1	10	16	+	10.8143	9.74e-05	0.253	CTTCCTG
1	mm10_knownGene_uc007rtb.2	4	10	-	10.8143	9.74e-05	0.253	CTTCCTG
1	mm10_knownGene_uc008xwd.2	10	16	+	10.8143	9.74e-05	0.253	CTTCCTG
1	mm10_knownGene_uc008lez.2	4	10	-	10.8143	9.74e-05	0.253	CTCCCTG
1	mm10_knownGene_uc009rjg.1	4	10	-	10.8143	9.74e-05	0.253	CTCCCTG
1	mm10_knownGene_uc009npe.1	10	16	+	10.8143	9.74e-05	0.253	CTTCCTG
1	mm10_knownGene_uc007jad.2	10	16	+	10.8143	9.74e-05	0.253	CTCCCTG
1	mm10_knownGene_uc056zap.1	10	16	+	10.8143	9.74e-05	0.253	CTCCCTG
1	mm10_knownGene_uc033ifm.1	4	10	-	10.8143	9.74e-05	0.253	CTTCCTG
1	mm10_knownGene_uc007jkj.1	10	16	+	10.8143	9.74e-05	0.253	CTCCCTG
1	mm10_knownGene_uc008xwc.2	10	16	+	10.8143	9.74e-05	0.253	CTTCCTG
1	mm10_knownGene_uc007rtc.2	4	10	-	10.8143	9.74e-05	0.253	CTTCCTG
1	mm10_knownGene_uc008uyh.2	4	10	-	10.8143	9.74e-05	0.253	CTTCCTG
1	mm10_knownGene_uc007rmd.2	10	16	+	10.8143	9.74e-05	0.253	CTCCCTG
1	mm10_knownGene_uc007fsw.2	4	10	-	10.8143	9.74e-05	0.253	CTCCCTG
1	mm10_knownGene_uc008qpg.1	4	10	-	10.8143	9.74e-05	0.253	CTTCCTG

1	mm10_knownGene_uc009ufn.1	5	11	-	10.8143	9.74e-05	0.253	CTTCCTG
1	mm10_knownGene_uc008fta.2	5	11	-	10.8143	9.74e-05	0.253	CTTCCTG
1	mm10_knownGene_uc011zds.1	11	17	+	10.8143	9.74e-05	0.253	CTTCCTG
1	mm10_knownGene_uc009mdb.2	5	11	-	10.8143	9.74e-05	0.253	CTTCCTG
1	mm10_knownGene_uc007kva.2	11	17	+	10.8143	9.74e-05	0.253	CTTCCTG
1	mm10_knownGene_uc007npb.1	11	17	+	10.8143	9.74e-05	0.253	CTTCCTG
1	mm10_knownGene_uc009hdb.3	11	17	+	10.8143	9.74e-05	0.253	CTTCCTG
1	mm10_knownGene_uc029qtb.1	11	17	+	10.8143	9.74e-05	0.253	CTTCCTG
1	mm10_knownGene_uc033fvf.1	11	17	+	10.8143	9.74e-05	0.253	CTTCCTG
1	mm10_knownGene_uc011zxd.1	5	11	-	10.8143	9.74e-05	0.253	CTTCCTG
1	mm10_knownGene_uc008zfx.4	5	11	-	10.8143	9.74e-05	0.253	CTTCCTG
1	mm10_knownGene_uc012flh.2	11	17	+	10.8143	9.74e-05	0.253	CTTCCTG
1	mm10_knownGene_uc009mii.1	5	11	-	10.8143	9.74e-05	0.253	CTCCCTG
1	mm10_knownGene_uc012bjx.1	5	11	-	10.8143	9.74e-05	0.253	CTTCCTG
1	mm10_knownGene_uc029vye.1	5	11	-	10.8143	9.74e-05	0.253	CTCCCTG
1	mm10_knownGene_uc009rhb.2	5	11	-	10.8143	9.74e-05	0.253	CTTCCTG
1	mm10_knownGene_uc012esq.1	11	17	+	10.8143	9.74e-05	0.253	CTTCCTG
1	mm10_knownGene_uc033ijt.1	11	17	+	10.8143	9.74e-05	0.253	CTCCCTG
1	mm10_knownGene_uc007lgm.1	5	11	-	10.8143	9.74e-05	0.253	CTTCCTG
1	mm10_knownGene_uc007rqo.1	11	17	+	10.8143	9.74e-05	0.253	CTTCCTG
1	mm10_knownGene_uc029syd.1	5	11	-	10.8143	9.74e-05	0.253	CTTCCTG
1	mm10_knownGene_uc007rqn.1	11	17	+	10.8143	9.74e-05	0.253	CTTCCTG
1	mm10_knownGene_uc008spq.2	11	17	+	10.8143	9.74e-05	0.253	CTCCCTG
1	mm10_knownGene_uc009ckv.2	11	17	+	10.8143	9.74e-05	0.253	CTTCCTG
1	mm10_knownGene_uc009sbe.2	11	17	+	10.8143	9.74e-05	0.253	CTCCCTG
1	mm10_knownGene_uc007fff.1	11	17	+	10.8143	9.74e-05	0.253	CTTCCTG
1	mm10_knownGene_uc007lgn.1	5	11	-	10.8143	9.74e-05	0.253	CTTCCTG
1	mm10_knownGene_uc009mih.2	5	11	-	10.8143	9.74e-05	0.253	CTCCCTG
1	mm10_knownGene_uc009erk.1	11	17	+	10.8143	9.74e-05	0.253	CTCCCTG
1	mm10_knownGene_uc009rhc.2	5	11	-	10.8143	9.74e-05	0.253	CTTCCTG
1	mm10_knownGene_uc009oww.1	5	11	-	10.8143	9.74e-05	0.253	CTCCCTG

1	mm10_knownGene_uc007dup.1	11	17	+	10.8143	9.74e-05	0.253	CTTCCTG
1	mm10_knownGene_uc007oqx.1	11	17	+	10.8143	9.74e-05	0.253	CTTCCTG
1	mm10_knownGene_uc008fsz.2	5	11	-	10.8143	9.74e-05	0.253	CTTCCTG
1	mm10_knownGene_uc009mig.1	5	11	-	10.8143	9.74e-05	0.253	CTCCCTG
1	mm10_knownGene_uc007xbh.2	5	11	-	10.8143	9.74e-05	0.253	CTTCCTG
1	mm10_knownGene_uc007zbj.1	5	11	-	10.8143	9.74e-05	0.253	CTCCCTG
1	mm10_knownGene_uc033gia.1	11	17	+	10.8143	9.74e-05	0.253	CTTCCTG
1	mm10_knownGene_uc009dsu.3	11	17	+	10.8143	9.74e-05	0.253	CTTCCTG
1	mm10_knownGene_uc012bfi.1	5	11	-	10.8143	9.74e-05	0.253	CTTCCTG
1	mm10_knownGene_uc007tnu.1	5	11	-	10.8143	9.74e-05	0.253	CTTCCTG
1	mm10_knownGene_uc033iss.1	11	17	+	10.8143	9.74e-05	0.253	CTTCCTG
1	mm10_knownGene_uc009jbs.1	5	11	-	10.8143	9.74e-05	0.253	CTCCCTG
1	mm10_knownGene_uc007pqg.2	5	11	-	10.8143	9.74e-05	0.253	CTTCCTG
1	mm10_knownGene_uc009htb.2	5	11	-	10.8143	9.74e-05	0.253	CTCCCTG
1	mm10_knownGene_uc007ffe.1	11	17	+	10.8143	9.74e-05	0.253	CTTCCTG
1	mm10_knownGene_uc012fli.2	11	17	+	10.8143	9.74e-05	0.253	CTTCCTG
1	mm10_knownGene_uc007pqh.2	5	11	-	10.8143	9.74e-05	0.253	CTTCCTG
1	mm10_knownGene_uc012gzj.1	5	11	-	10.8143	9.74e-05	0.253	CTTCCTG
1	mm10_knownGene_uc009cqy.2	11	17	+	10.8143	9.74e-05	0.253	CTTCCTG
1	mm10_knownGene_uc009hdc.3	11	17	+	10.8143	9.74e-05	0.253	CTTCCTG
1	mm10_knownGene_uc007lzd.1	12	18	+	10.8143	9.74e-05	0.253	CTTCCTG
1	mm10_knownGene_uc009mqb.1	6	12	-	10.8143	9.74e-05	0.253	CTTCCTG
1	mm10_knownGene_uc008rem.1	12	18	+	10.8143	9.74e-05	0.253	CTTCCTG
1	mm10_knownGene_uc009fjm.1	6	12	-	10.8143	9.74e-05	0.253	CTCCCTG
1	mm10_knownGene_uc007ecg.1	6	12	-	10.8143	9.74e-05	0.253	CTTCCTG
1	mm10_knownGene_uc007hyw.2	6	12	-	10.8143	9.74e-05	0.253	CTTCCTG
1	mm10_knownGene_uc009kbz.1	12	18	+	10.8143	9.74e-05	0.253	CTTCCTG
1	mm10_knownGene_uc008qze.2	12	18	+	10.8143	9.74e-05	0.253	CTCCCTG
1	mm10_knownGene_uc012cgb.1	6	12	-	10.8143	9.74e-05	0.253	CTTCCTG
1	mm10_knownGene_uc008xvh.2	6	12	-	10.8143	9.74e-05	0.253	CTTCCTG
1	mm10_knownGene_uc008yhf.1	12	18	+	10.8143	9.74e-05	0.253	CTCCCTG

1	mm10_knownGene_uc008zgn.1	6	12	-	10.8143	9.74e-05	0.253	CTTCCTG
1	mm10_knownGene_uc009aco.1	12	18	+	10.8143	9.74e-05	0.253	CTTCCTG
1	mm10_knownGene_uc009bsa.1	12	18	+	10.8143	9.74e-05	0.253	CTTCCTG
1	mm10_knownGene_uc008zjx.1	12	18	+	10.8143	9.74e-05	0.253	CTCCCTG
1	mm10_knownGene_uc009iup.1	6	12	-	10.8143	9.74e-05	0.253	CTTCCTG
1	mm10_knownGene_uc011zsr.2	6	12	-	10.8143	9.74e-05	0.253	CTCCCTG
1	mm10_knownGene_uc009anc.2	12	18	+	10.8143	9.74e-05	0.253	CTCCCTG
1	mm10_knownGene_uc008hvy.1	6	12	-	10.8143	9.74e-05	0.253	CTCCCTG
1	mm10_knownGene_uc008zjv.2	12	18	+	10.8143	9.74e-05	0.253	CTCCCTG
1	mm10_knownGene_uc011ytm.1	6	12	-	10.8143	9.74e-05	0.253	CTTCCTG
1	mm10_knownGene_uc007rjf.2	12	18	+	10.8143	9.74e-05	0.253	CTCCCTG
1	mm10_knownGene_uc009qtn.2	12	18	+	10.8143	9.74e-05	0.253	CTTCCTG
1	mm10_knownGene_uc009mll.3	6	12	-	10.8143	9.74e-05	0.253	CTCCCTG
1	mm10_knownGene_uc009pqu.2	12	18	+	10.8143	9.74e-05	0.253	CTTCCTG
1	mm10_knownGene_uc012bma.1	12	18	+	10.8143	9.74e-05	0.253	CTTCCTG
1	mm10_knownGene_uc008jsq.1	6	12	-	10.8143	9.74e-05	0.253	CTTCCTG
1	mm10_knownGene_uc008hoh.2	12	18	+	10.8143	9.74e-05	0.253	CTTCCTG
1	mm10_knownGene_uc012hjf.1	12	18	+	10.8143	9.74e-05	0.253	CTCCCTG
1	mm10_knownGene_uc008ivm.1	12	18	+	10.8143	9.74e-05	0.253	CTTCCTG
1	mm10_knownGene_uc012cmh.1	6	12	-	10.8143	9.74e-05	0.253	CTCCCTG
1	mm10_knownGene_uc009rxo.2	7	13	-	10.8143	9.74e-05	0.253	CTTCCTG
1	mm10_knownGene_uc033ilo.1	7	13	-	10.8143	9.74e-05	0.253	CTCCCTG
1	mm10_knownGene_uc007frh.2	13	19	+	10.8143	9.74e-05	0.253	CTTCCTG
1	mm10_knownGene_uc012bdk.1	13	19	+	10.8143	9.74e-05	0.253	CTTCCTG
1	mm10_knownGene_uc009cru.2	7	13	-	10.8143	9.74e-05	0.253	CTCCCTG
1	mm10_knownGene_uc033iys.1	7	13	-	10.8143	9.74e-05	0.253	CTTCCTG
1	mm10_knownGene_uc029tdb.1	7	13	-	10.8143	9.74e-05	0.253	CTTCCTG
1	mm10_knownGene_uc007jet.2	13	19	+	10.8143	9.74e-05	0.253	CTTCCTG
1	mm10_knownGene_uc007dql.2	7	13	-	10.8143	9.74e-05	0.253	CTTCCTG
1	mm10_knownGene_uc011wjc.1	13	19	+	10.8143	9.74e-05	0.253	CTCCCTG
1	mm10_knownGene_uc033gqs.1	7	13	-	10.8143	9.74e-05	0.253	CTCCCTG

1	mm10_knownGene_uc033foa.1	13	19	+	10.8143	9.74e-05	0.253	CTTCCTG
1	mm10_knownGene_uc011zvl.1	7	13	-	10.8143	9.74e-05	0.253	CTCCCTG
1	mm10_knownGene_uc007fnx.2	7	13	-	10.8143	9.74e-05	0.253	CTTCCTG
1	mm10_knownGene_uc029qnh.1	7	13	-	10.8143	9.74e-05	0.253	CTCCCTG
1	mm10_knownGene_uc007ejj.2	7	13	-	10.8143	9.74e-05	0.253	CTTCCTG
1	mm10_knownGene_uc007hzj.1	7	13	-	10.8143	9.74e-05	0.253	CTCCCTG
1	mm10_knownGene_uc007cai.1	13	19	+	10.8143	9.74e-05	0.253	CTTCCTG
1	mm10_knownGene_uc008sun.2	7	13	-	10.8143	9.74e-05	0.253	CTTCCTG
1	mm10_knownGene_uc033jux.1	7	13	-	10.8143	9.74e-05	0.253	CTTCCTG
1	mm10_knownGene_uc012axf.1	13	19	+	10.8143	9.74e-05	0.253	CTTCCTG
1	mm10_knownGene_uc012gkf.1	7	13	-	10.8143	9.74e-05	0.253	CTTCCTG
1	mm10_knownGene_uc007fny.2	7	13	-	10.8143	9.74e-05	0.253	CTTCCTG
1	mm10_knownGene_uc033ful.1	13	19	+	10.8143	9.74e-05	0.253	CTCCCTG
1	mm10_knownGene_uc033fvi.1	13	19	+	10.8143	9.74e-05	0.253	CTCCCTG
1	mm10_knownGene_uc007khv.1	7	13	-	10.8143	9.74e-05	0.253	CTTCCTG
1	mm10_knownGene_uc009pph.2	7	13	-	10.8143	9.74e-05	0.253	CTTCCTG
1	mm10_knownGene_uc008aad.2	13	19	+	10.8143	9.74e-05	0.253	CTCCCTG
1	mm10_knownGene_uc007fnw.2	7	13	-	10.8143	9.74e-05	0.253	CTTCCTG
1	mm10_knownGene_uc008uxy.2	13	19	+	10.8143	9.74e-05	0.253	CTCCCTG
1	mm10_knownGene_uc008vdp.1	7	13	-	10.8143	9.74e-05	0.253	CTTCCTG
1	mm10_knownGene_uc008suo.2	7	13	-	10.8143	9.74e-05	0.253	CTTCCTG
1	mm10_knownGene_uc007eji.2	7	13	-	10.8143	9.74e-05	0.253	CTTCCTG
1	mm10_knownGene_uc009aae.2	14	20	+	10.8143	9.74e-05	0.253	CTTCCTG
1	mm10_knownGene_uc008jni.2	8	14	-	10.8143	9.74e-05	0.253	CTCCCTG
1	mm10_knownGene_uc007arb.2	8	14	-	10.8143	9.74e-05	0.253	CTCCCTG
1	mm10_knownGene_uc008jvm.2	8	14	-	10.8143	9.74e-05	0.253	CTTCCTG
1	mm10_knownGene_uc012eoj.2	8	14	-	10.8143	9.74e-05	0.253	CTCCCTG
1	mm10_knownGene_uc009jxl.1	14	20	+	10.8143	9.74e-05	0.253	CTCCCTG
1	mm10_knownGene_uc012gon.3	14	20	+	10.8143	9.74e-05	0.253	CTTCCTG
1	mm10_knownGene_uc033hpt.1	14	20	+	10.8143	9.74e-05	0.253	CTCCCTG
1	mm10_knownGene_uc033fpc.1	14	20	+	10.8143	9.74e-05	0.253	CTTCCTG

1 mm10_knownGene_uc008gar.1 8 14 - 10.8143 9.74e-
05 0.253 CTTCCTG
1 mm10_knownGene_uc009svg.1 8 14 - 10.8143 9.74e-
05 0.253 CTCCCTG
1 mm10_knownGene_uc008nju.2 8 14 - 10.8143 9.74e-
05 0.253 CTCCCTG
1 mm10_knownGene_uc008njr.2 8 14 - 10.8143 9.74e-
05 0.253 CTCCCTG
1 mm10_knownGene_uc009gmn.1 14 20 + 10.8143 9.74e-
05 0.253 CTTCCTG
1 mm10_knownGene_uc008njs.2 8 14 - 10.8143 9.74e-
05 0.253 CTCCCTG
1 mm10_knownGene_uc009jxk.1 14 20 + 10.8143 9.74e-
05 0.253 CTCCCTG
1 mm10_knownGene_uc008usu.1 14 20 + 10.8143 9.74e-
05 0.253 CTCCCTG
1 mm10_knownGene_uc009flz.1 8 14 - 10.8143 9.74e-
05 0.253 CTTCCTG
1 mm10_knownGene_uc008njv.2 8 14 - 10.8143 9.74e-
05 0.253 CTCCCTG
1 mm10_knownGene_uc008djc.1 14 20 + 10.8143 9.74e-
05 0.253 CTTCCTG
1 mm10_knownGene_uc008cci.1 14 20 + 10.8143 9.74e-
05 0.253 CTCCCTG
1 mm10_knownGene_uc008jng.2 8 14 - 10.8143 9.74e-
05 0.253 CTCCCTG
1 mm10_knownGene_uc007dvi.1 8 14 - 10.8143 9.74e-
05 0.253 CTCCCTG
1 mm10_knownGene_uc009aad.2 14 20 + 10.8143 9.74e-
05 0.253 CTTCCTG
1 mm10_knownGene_uc008obp.2 14 20 + 10.8143 9.74e-
05 0.253 CTCCCTG
1 mm10_knownGene_uc008gas.2 8 14 - 10.8143 9.74e-
05 0.253 CTTCCTG
1 mm10_knownGene_uc008obo.2 14 20 + 10.8143 9.74e-
05 0.253 CTCCCTG
1 mm10_knownGene_uc012bvy.1 8 14 - 10.8143 9.74e-
05 0.253 CTTCCTG
1 mm10_knownGene_uc008uhe.1 8 14 - 10.8143 9.74e-
05 0.253 CTTCCTG
1 mm10_knownGene_uc008gaq.1 8 14 - 10.8143 9.74e-
05 0.253 CTTCCTG
1 mm10_knownGene_uc012eao.1 14 20 + 10.8143 9.74e-
05 0.253 CTTCCTG
1 mm10_knownGene_uc012bgk.1 8 14 - 10.8143 9.74e-
05 0.253 CTTCCTG
1 mm10_knownGene_uc007muk.1 14 20 + 10.8143 9.74e-
05 0.253 CTTCCTG
1 mm10_knownGene_uc008yxl.1 14 20 + 10.8143 9.74e-
05 0.253 CTCCCTG
1 mm10_knownGene_uc009ofi.1 14 20 + 10.8143 9.74e-
05 0.253 CTTCCTG
1 mm10_knownGene_uc008jvo.2 8 14 - 10.8143 9.74e-
05 0.253 CTTCCTG
1 mm10_knownGene_uc009iju.1 8 14 - 10.8143 9.74e-
05 0.253 CTTCCTG
1 mm10_knownGene_uc009qic.1 14 20 + 10.8143 9.74e-
05 0.253 CTCCCTG
1 mm10_knownGene_uc008njt.2 8 14 - 10.8143 9.74e-
05 0.253 CTCCCTG
1 mm10_knownGene_uc057ccp.1 14 20 + 10.8143 9.74e-
05 0.253 CTTCCTG

1	mm10_knownGene_uc008dul.1	8	14	-	10.8143	9.74e-05	0.253	CTTCCTG
1	mm10_knownGene_uc056yob.1	14	20	+	10.8143	9.74e-05	0.253	CTTCCTG
1	mm10_knownGene_uc008jnh.2	8	14	-	10.8143	9.74e-05	0.253	CTCCCTG
1	mm10_knownGene_uc008mxt.1	14	20	+	10.8143	9.74e-05	0.253	CTTCCTG
1	mm10_knownGene_uc008jvn.2	8	14	-	10.8143	9.74e-05	0.253	CTTCCTG
1	mm10_knownGene_uc033izh.1	8	14	-	10.8143	9.74e-05	0.253	CTCCCTG
1	mm10_knownGene_uc008dzn.1	14	20	+	10.8143	9.74e-05	0.253	CTTCCTG
1	mm10_knownGene_uc012cgy.1	8	14	-	10.8143	9.74e-05	0.253	CTCCCTG
1	mm10_knownGene_uc029uwx.1	14	20	+	10.8143	9.74e-05	0.253	CTCCCTG
1	mm10_knownGene_uc009kih.1	8	14	-	10.8143	9.74e-05	0.253	CTCCCTG
1	mm10_knownGene_uc008vkw.1	15	21	+	10.8143	9.74e-05	0.253	CTCCCTG
1	mm10_knownGene_uc029ugt.1	15	21	+	10.8143	9.74e-05	0.253	CTTCCTG
1	mm10_knownGene_uc008yjy.1	15	21	+	10.8143	9.74e-05	0.253	CTTCCTG
1	mm10_knownGene_uc029rzw.1	9	15	-	10.8143	9.74e-05	0.253	CTCCCTG
1	mm10_knownGene_uc008nbz.1	15	21	+	10.8143	9.74e-05	0.253	CTTCCTG
1	mm10_knownGene_uc008bxy.1	9	15	-	10.8143	9.74e-05	0.253	CTTCCTG
1	mm10_knownGene_uc009tqk.2	9	15	-	10.8143	9.74e-05	0.253	CTTCCTG
1	mm10_knownGene_uc009edh.1	9	15	-	10.8143	9.74e-05	0.253	CTCCCTG
1	mm10_knownGene_uc008asa.1	15	21	+	10.8143	9.74e-05	0.253	CTTCCTG
1	mm10_knownGene_uc033hvd.1	15	21	+	10.8143	9.74e-05	0.253	CTTCCTG
1	mm10_knownGene_uc008nfj.2	9	15	-	10.8143	9.74e-05	0.253	CTCCCTG
1	mm10_knownGene_uc012ccx.1	9	15	-	10.8143	9.74e-05	0.253	CTTCCTG
1	mm10_knownGene_uc007xyy.1	15	21	+	10.8143	9.74e-05	0.253	CTCCCTG
1	mm10_knownGene_uc008yjz.1	15	21	+	10.8143	9.74e-05	0.253	CTTCCTG
1	mm10_knownGene_uc009kpd.2	15	21	+	10.8143	9.74e-05	0.253	CTCCCTG
1	mm10_knownGene_uc056ykq.1	15	21	+	10.8143	9.74e-05	0.253	CTCCCTG
1	mm10_knownGene_uc009aaf.1	15	21	+	10.8143	9.74e-05	0.253	CTCCCTG
1	mm10_knownGene_uc057bvd.1	9	15	-	10.8143	9.74e-05	0.253	CTTCCTG
1	mm10_knownGene_uc009lkw.1	9	15	-	10.8143	9.74e-05	0.253	CTTCCTG
1	mm10_knownGene_uc009qdu.2	15	21	+	10.8143	9.74e-05	0.253	CTTCCTG
1	mm10_knownGene_uc029vpb.1	15	21	+	10.8143	9.74e-05	0.253	CTTCCTG

1	mm10_knownGene_uc007dys.1	9	15	-	10.8143	9.74e-05	0.253	CTTCCTG
1	mm10_knownGene_uc007uqv.1	9	15	-	10.8143	9.74e-05	0.253	CTCCCTG
1	mm10_knownGene_uc012faq.1	9	15	-	10.8143	9.74e-05	0.253	CTTCCTG
1	mm10_knownGene_uc007ilm.1	9	15	-	10.8143	9.74e-05	0.253	CTCCCTG
1	mm10_knownGene_uc008mak.1	9	15	-	10.8143	9.74e-05	0.253	CTTCCTG
1	mm10_knownGene_uc033ivq.1	15	21	+	10.8143	9.74e-05	0.253	CTTCCTG
1	mm10_knownGene_uc009lwv.1	15	21	+	10.8143	9.74e-05	0.253	CTCCCTG
1	mm10_knownGene_uc009edi.1	9	15	-	10.8143	9.74e-05	0.253	CTCCCTG
1	mm10_knownGene_uc009kpe.2	15	21	+	10.8143	9.74e-05	0.253	CTCCCTG
1	mm10_knownGene_uc029vmx.1	9	15	-	10.8143	9.74e-05	0.253	CTCCCTG
1	mm10_knownGene_uc011xwu.1	9	15	-	10.8143	9.74e-05	0.253	CTCCCTG
1	mm10_knownGene_uc057aox.1	15	21	+	10.8143	9.74e-05	0.253	CTTCCTG
1	mm10_knownGene_uc009kpf.2	15	21	+	10.8143	9.74e-05	0.253	CTCCCTG
1	mm10_knownGene_uc008koy.1	9	15	-	10.8143	9.74e-05	0.253	CTCCCTG
1	mm10_knownGene_uc012hnz.1	15	21	+	10.8143	9.74e-05	0.253	CTCCCTG
1	mm10_knownGene_uc029vuk.1	15	21	+	10.8143	9.74e-05	0.253	CTTCCTG
1	mm10_knownGene_uc009eoj.2	10	16	-	10.8143	9.74e-05	0.253	CTTCCTG
1	mm10_knownGene_uc012cjh.1	10	16	-	10.8143	9.74e-05	0.253	CTTCCTG
1	mm10_knownGene_uc029wcf.1	10	16	-	10.8143	9.74e-05	0.253	CTCCCTG
1	mm10_knownGene_uc029qsk.1	10	16	-	10.8143	9.74e-05	0.253	CTTCCTG
1	mm10_knownGene_uc008ota.1	16	22	+	10.8143	9.74e-05	0.253	CTTCCTG
1	mm10_knownGene_uc007unl.1	10	16	-	10.8143	9.74e-05	0.253	CTTCCTG
1	mm10_knownGene_uc008kgm.1	16	22	+	10.8143	9.74e-05	0.253	CTTCCTG
1	mm10_knownGene_uc008ooh.2	10	16	-	10.8143	9.74e-05	0.253	CTCCCTG
1	mm10_knownGene_uc007qbl.1	16	22	+	10.8143	9.74e-05	0.253	CTTCCTG
1	mm10_knownGene_uc007orm.1	16	22	+	10.8143	9.74e-05	0.253	CTTCCTG
1	mm10_knownGene_uc008kgn.1	16	22	+	10.8143	9.74e-05	0.253	CTTCCTG
1	mm10_knownGene_uc009psn.1	10	16	-	10.8143	9.74e-05	0.253	CTCCCTG
1	mm10_knownGene_uc007bnc.1	10	16	-	10.8143	9.74e-05	0.253	CTCCCTG
1	mm10_knownGene_uc012fic.2	10	16	-	10.8143	9.74e-05	0.253	CTTCCTG
1	mm10_knownGene_uc009aif.1	16	22	+	10.8143	9.74e-05	0.253	CTCCCTG

1	mm10_knownGene_uc033gqr.1	16	22	+	10.8143	9.74e-05	0.253	CTCCCTG
1	mm10_knownGene_uc007fnz.2	10	16	-	10.8143	9.74e-05	0.253	CTCCCTG
1	mm10_knownGene_uc007cpw.2	10	16	-	10.8143	9.74e-05	0.253	CTCCCTG
1	mm10_knownGene_uc007fob.2	10	16	-	10.8143	9.74e-05	0.253	CTCCCTG
1	mm10_knownGene_uc008jkv.2	10	16	-	10.8143	9.74e-05	0.253	CTTCCTG
1	mm10_knownGene_uc011yfg.1	10	16	-	10.8143	9.74e-05	0.253	CTCCCTG
1	mm10_knownGene_uc033gtx.1	10	16	-	10.8143	9.74e-05	0.253	CTTCCTG
1	mm10_knownGene_uc009eok.2	10	16	-	10.8143	9.74e-05	0.253	CTTCCTG
1	mm10_knownGene_uc009cuo.2	10	16	-	10.8143	9.74e-05	0.253	CTTCCTG
1	mm10_knownGene_uc008ykg.2	10	16	-	10.8143	9.74e-05	0.253	CTCCCTG
1	mm10_knownGene_uc008fgp.2	10	16	-	10.8143	9.74e-05	0.253	CTTCCTG
1	mm10_knownGene_uc008egs.2	10	16	-	10.8143	9.74e-05	0.253	CTTCCTG
1	mm10_knownGene_uc007nrx.1	16	22	+	10.8143	9.74e-05	0.253	CTCCCTG
1	mm10_knownGene_uc011wrq.1	16	22	+	10.8143	9.74e-05	0.253	CTTCCTG
1	mm10_knownGene_uc007vmg.2	10	16	-	10.8143	9.74e-05	0.253	CTTCCTG
1	mm10_knownGene_uc007avb.1	10	16	-	10.8143	9.74e-05	0.253	CTTCCTG
1	mm10_knownGene_uc007lmn.1	10	16	-	10.8143	9.74e-05	0.253	CTCCCTG
1	mm10_knownGene_uc007foa.2	10	16	-	10.8143	9.74e-05	0.253	CTCCCTG
1	mm10_knownGene_uc029xkw.1	16	22	+	10.8143	9.74e-05	0.253	CTTCCTG
1	mm10_knownGene_uc007fjy.2	10	16	-	10.8143	9.74e-05	0.253	CTTCCTG
1	mm10_knownGene_uc029wre.1	10	16	-	10.8143	9.74e-05	0.253	CTTCCTG
1	mm10_knownGene_uc008sil.2	10	16	-	10.8143	9.74e-05	0.253	CTCCCTG
1	mm10_knownGene_uc009gwt.2	16	22	+	10.8143	9.74e-05	0.253	CTCCCTG
1	mm10_knownGene_uc008jkw.1	10	16	-	10.8143	9.74e-05	0.253	CTTCCTG
1	mm10_knownGene_uc009btu.1	11	17	-	10.8143	9.74e-05	0.253	CTTCCTG
1	mm10_knownGene_uc007leb.1	17	23	+	10.8143	9.74e-05	0.253	CTTCCTG
1	mm10_knownGene_uc009mqz.2	17	23	+	10.8143	9.74e-05	0.253	CTCCCTG
1	mm10_knownGene_uc007pjz.2	11	17	-	10.8143	9.74e-05	0.253	CTTCCTG
1	mm10_knownGene_uc009mrb.2	17	23	+	10.8143	9.74e-05	0.253	CTCCCTG
1	mm10_knownGene_uc009lat.2	11	17	-	10.8143	9.74e-05	0.253	CTTCCTG
1	mm10_knownGene_uc008nsc.2	17	23	+	10.8143	9.74e-05	0.253	CTCCCTG

1	mm10_knownGene_uc008pty.1	17	23	+	10.8143	9.74e-05	0.253	CTTCCTG
1	mm10_knownGene_uc012clz.1	11	17	-	10.8143	9.74e-05	0.253	CTTCCTG
1	mm10_knownGene_uc007pkd.2	11	17	-	10.8143	9.74e-05	0.253	CTTCCTG
1	mm10_knownGene_uc009gsj.2	11	17	-	10.8143	9.74e-05	0.253	CTCCCTG
1	mm10_knownGene_uc007kxa.1	11	17	-	10.8143	9.74e-05	0.253	CTCCCTG
1	mm10_knownGene_uc007qoz.2	17	23	+	10.8143	9.74e-05	0.253	CTTCCTG
1	mm10_knownGene_uc008bsm.1	11	17	-	10.8143	9.74e-05	0.253	CTTCCTG
1	mm10_knownGene_uc007pka.2	11	17	-	10.8143	9.74e-05	0.253	CTTCCTG
1	mm10_knownGene_uc007sqx.1	17	23	+	10.8143	9.74e-05	0.253	CTCCCTG
1	mm10_knownGene_uc009mqx.2	17	23	+	10.8143	9.74e-05	0.253	CTCCCTG
1	mm10_knownGene_uc009ddr.1	17	23	+	10.8143	9.74e-05	0.253	CTCCCTG
1	mm10_knownGene_uc007omd.2	11	17	-	10.8143	9.74e-05	0.253	CTTCCTG
1	mm10_knownGene_uc009mra.2	17	23	+	10.8143	9.74e-05	0.253	CTCCCTG
1	mm10_knownGene_uc009ddq.1	17	23	+	10.8143	9.74e-05	0.253	CTCCCTG
1	mm10_knownGene_uc007gch.1	17	23	+	10.8143	9.74e-05	0.253	CTTCCTG
1	mm10_knownGene_uc007pkc.2	11	17	-	10.8143	9.74e-05	0.253	CTTCCTG
1	mm10_knownGene_uc009hcy.1	17	23	+	10.8143	9.74e-05	0.253	CTTCCTG
1	mm10_knownGene_uc008oiw.1	11	17	-	10.8143	9.74e-05	0.253	CTTCCTG
1	mm10_knownGene_uc009hcz.2	17	23	+	10.8143	9.74e-05	0.253	CTTCCTG
1	mm10_knownGene_uc007lia.1	11	17	-	10.8143	9.74e-05	0.253	CTTCCTG
1	mm10_knownGene_uc008oiv.1	11	17	-	10.8143	9.74e-05	0.253	CTTCCTG
1	mm10_knownGene_uc011xin.1	17	23	+	10.8143	9.74e-05	0.253	CTTCCTG
1	mm10_knownGene_uc009len.1	11	17	-	10.8143	9.74e-05	0.253	CTTCCTG
1	mm10_knownGene_uc008ptx.1	17	23	+	10.8143	9.74e-05	0.253	CTTCCTG
1	mm10_knownGene_uc007tuu.1	17	23	+	10.8143	9.74e-05	0.253	CTTCCTG
1	mm10_knownGene_uc029uop.1	11	17	-	10.8143	9.74e-05	0.253	CTTCCTG
1	mm10_knownGene_uc007rbt.2	11	17	-	10.8143	9.74e-05	0.253	CTTCCTG
1	mm10_knownGene_uc007kdy.1	18	24	+	10.8143	9.74e-05	0.253	CTCCCTG
1	mm10_knownGene_uc033hdc.1	18	24	+	10.8143	9.74e-05	0.253	CTTCCTG
1	mm10_knownGene_uc008fit.2	18	24	+	10.8143	9.74e-05	0.253	CTTCCTG
1	mm10_knownGene_uc033hde.1	18	24	+	10.8143	9.74e-05	0.253	CTTCCTG

1	mm10_knownGene_uc009ffk.1	18	24	+	10.8143	9.74e-05	0.253	CTCCCTG
1	mm10_knownGene_uc007ufh.3	18	24	+	10.8143	9.74e-05	0.253	CTCCCTG
1	mm10_knownGene_uc011zbp.1	18	24	+	10.8143	9.74e-05	0.253	CTCCCTG
1	mm10_knownGene_uc029trd.1	12	18	-	10.8143	9.74e-05	0.253	CTCCCTG
1	mm10_knownGene_uc009pgu.2	18	24	+	10.8143	9.74e-05	0.253	CTTCCTG
1	mm10_knownGene_uc033gtr.1	12	18	-	10.8143	9.74e-05	0.253	CTCCCTG
1	mm10_knownGene_uc008edj.2	12	18	-	10.8143	9.74e-05	0.253	CTCCCTG
1	mm10_knownGene_uc033hdd.1	18	24	+	10.8143	9.74e-05	0.253	CTTCCTG
1	mm10_knownGene_uc009thu.3	18	24	+	10.8143	9.74e-05	0.253	CTCCCTG
1	mm10_knownGene_uc033gtu.1	12	18	-	10.8143	9.74e-05	0.253	CTCCCTG
1	mm10_knownGene_uc012azs.1	12	18	-	10.8143	9.74e-05	0.253	CTCCCTG
1	mm10_knownGene_uc007unt.1	18	24	+	10.8143	9.74e-05	0.253	CTTCCTG
1	mm10_knownGene_uc009eyy.2	12	18	-	10.8143	9.74e-05	0.253	CTTCCTG
1	mm10_knownGene_uc009pbw.2	18	24	+	10.8143	9.74e-05	0.253	CTTCCTG
1	mm10_knownGene_uc009fvx.1	18	24	+	10.8143	9.74e-05	0.253	CTCCCTG
1	mm10_knownGene_uc011zbq.1	18	24	+	10.8143	9.74e-05	0.253	CTCCCTG
1	mm10_knownGene_uc008mer.1	18	24	+	10.8143	9.74e-05	0.253	CTTCCTG
1	mm10_knownGene_uc007unu.1	18	24	+	10.8143	9.74e-05	0.253	CTTCCTG
1	mm10_knownGene_uc009rzd.1	18	24	+	10.8143	9.74e-05	0.253	CTCCCTG
1	mm10_knownGene_uc008onr.2	18	24	+	10.8143	9.74e-05	0.253	CTTCCTG
1	mm10_knownGene_uc029vww.1	12	18	-	10.8143	9.74e-05	0.253	CTCCCTG
1	mm10_knownGene_uc009pgw.1	18	24	+	10.8143	9.74e-05	0.253	CTTCCTG
1	mm10_knownGene_uc009kku.1	18	24	+	10.8143	9.74e-05	0.253	CTCCCTG
1	mm10_knownGene_uc009dvv.1	18	24	+	10.8143	9.74e-05	0.253	CTCCCTG
1	mm10_knownGene_uc011xxy.1	18	24	+	10.8143	9.74e-05	0.253	CTTCCTG
1	mm10_knownGene_uc033gtt.1	12	18	-	10.8143	9.74e-05	0.253	CTCCCTG
1	mm10_knownGene_uc008zen.1	18	24	+	10.8143	9.74e-05	0.253	CTTCCTG
1	mm10_knownGene_uc007unv.1	18	24	+	10.8143	9.74e-05	0.253	CTTCCTG
1	mm10_knownGene_uc008uun.2	18	24	+	10.8143	9.74e-05	0.253	CTTCCTG
1	mm10_knownGene_uc009pbx.2	18	24	+	10.8143	9.74e-05	0.253	CTTCCTG
1	mm10_knownGene_uc009fvw.1	18	24	+	10.8143	9.74e-05	0.253	CTCCCTG

1	mm10_knownGene_uc009fvy.1	18	24	+	10.8143	9.74e-05	0.253	CTCCCTG
1	mm10_knownGene_uc007ufg.3	18	24	+	10.8143	9.74e-05	0.253	CTCCCTG
1	mm10_knownGene_uc009pgv.1	18	24	+	10.8143	9.74e-05	0.253	CTTCCTG
1	mm10_knownGene_uc009pgt.2	18	24	+	10.8143	9.74e-05	0.253	CTTCCTG
1	mm10_knownGene_uc029via.1	18	24	+	10.8143	9.74e-05	0.253	CTTCCTG
1	mm10_knownGene_uc033gts.1	12	18	-	10.8143	9.74e-05	0.253	CTCCCTG
1	mm10_knownGene_uc008yve.1	12	18	-	10.8143	9.74e-05	0.253	CTCCCTG
1	mm10_knownGene_uc007lek.2	12	18	-	10.8143	9.74e-05	0.253	CTCCCTG
1	mm10_knownGene_uc012azr.1	12	18	-	10.8143	9.74e-05	0.253	CTCCCTG
1	mm10_knownGene_uc008zkl.1	18	24	+	10.8143	9.74e-05	0.253	CTCCCTG
1	mm10_knownGene_uc033hej.1	18	24	+	10.8143	9.74e-05	0.253	CTCCCTG
1	mm10_knownGene_uc007hyk.1	13	19	-	10.8143	9.74e-05	0.253	CTTCCTG
1	mm10_knownGene_uc012dib.1	19	25	+	10.8143	9.74e-05	0.253	CTCCCTG
1	mm10_knownGene_uc009vdz.1	13	19	-	10.8143	9.74e-05	0.253	CTTCCTG
1	mm10_knownGene_uc008xlj.2	19	25	+	10.8143	9.74e-05	0.253	CTCCCTG
1	mm10_knownGene_uc007hzz.1	13	19	-	10.8143	9.74e-05	0.253	CTTCCTG
1	mm10_knownGene_uc007nyb.1	19	25	+	10.8143	9.74e-05	0.253	CTCCCTG
1	mm10_knownGene_uc007xpw.1	13	19	-	10.8143	9.74e-05	0.253	CTCCCTG
1	mm10_knownGene_uc008tzd.1	19	25	+	10.8143	9.74e-05	0.253	CTCCCTG
1	mm10_knownGene_uc007xpv.1	13	19	-	10.8143	9.74e-05	0.253	CTCCCTG
1	mm10_knownGene_uc008csd.2	19	25	+	10.8143	9.74e-05	0.253	CTCCCTG
1	mm10_knownGene_uc007vlp.2	13	19	-	10.8143	9.74e-05	0.253	CTCCCTG
1	mm10_knownGene_uc009lzr.4	13	19	-	10.8143	9.74e-05	0.253	CTCCCTG
1	mm10_knownGene_uc007iaa.1	13	19	-	10.8143	9.74e-05	0.253	CTTCCTG
1	mm10_knownGene_uc007feq.1	19	25	+	10.8143	9.74e-05	0.253	CTCCCTG
1	mm10_knownGene_uc009mce.1	19	25	+	10.8143	9.74e-05	0.253	CTTCCTG
1	mm10_knownGene_uc009frk.2	19	25	+	10.8143	9.74e-05	0.253	CTCCCTG
1	mm10_knownGene_uc009cob.1	19	25	+	10.8143	9.74e-05	0.253	CTTCCTG
1	mm10_knownGene_uc007duq.2	19	25	+	10.8143	9.74e-05	0.253	CTTCCTG
1	mm10_knownGene_uc007ddf.2	19	25	+	10.8143	9.74e-05	0.253	CTTCCTG
1	mm10_knownGene_uc012caj.1	13	19	-	10.8143	9.74e-05	0.253	CTCCCTG

1	mm10_knownGene_uc011yml.2	13	19	-	10.8143	9.74e-05	0.253	CTTCCTG
1	mm10_knownGene_uc029tsa.1	19	25	+	10.8143	9.74e-05	0.253	CTTCCTG
1	mm10_knownGene_uc056yej.1	19	25	+	10.8143	9.74e-05	0.253	CTTCCTG
1	mm10_knownGene_uc009lzs.2	13	19	-	10.8143	9.74e-05	0.253	CTCCCTG
1	mm10_knownGene_uc029qrr.1	19	25	+	10.8143	9.74e-05	0.253	CTTCCTG
1	mm10_knownGene_uc007jgu.2	19	25	+	10.8143	9.74e-05	0.253	CTTCCTG
1	mm10_knownGene_uc009lzt.2	13	19	-	10.8143	9.74e-05	0.253	CTCCCTG
1	mm10_knownGene_uc008yus.2	19	25	+	10.8143	9.74e-05	0.253	CTTCCTG
1	mm10_knownGene_uc008zov.1	13	19	-	10.8143	9.74e-05	0.253	CTTCCTG
1	mm10_knownGene_uc009hrf.2	19	25	+	10.8143	9.74e-05	0.253	CTCCCTG
1	mm10_knownGene_uc008iry.1	19	25	+	10.8143	9.74e-05	0.253	CTTCCTG
1	mm10_knownGene_uc009qle.2	19	25	+	10.8143	9.74e-05	0.253	CTTCCTG
1	mm10_knownGene_uc007gif.1	13	19	-	10.8143	9.74e-05	0.253	CTTCCTG
1	mm10_knownGene_uc007asx.1	13	19	-	10.8143	9.74e-05	0.253	CTTCCTG
1	mm10_knownGene_uc007dde.2	19	25	+	10.8143	9.74e-05	0.253	CTTCCTG
1	mm10_knownGene_uc007jvc.1	13	19	-	10.8143	9.74e-05	0.253	CTTCCTG
1	mm10_knownGene_uc056ztv.1	19	25	+	10.8143	9.74e-05	0.253	CTTCCTG
1	mm10_knownGene_uc012dxe.1	13	19	-	10.8143	9.74e-05	0.253	CTTCCTG
1	mm10_knownGene_uc008xfs.2	20	26	+	10.8143	9.74e-05	0.253	CTTCCTG
1	mm10_knownGene_uc029ssr.1	20	26	+	10.8143	9.74e-05	0.253	CTCCCTG
1	mm10_knownGene_uc056yot.1	14	20	-	10.8143	9.74e-05	0.253	CTTCCTG
1	mm10_knownGene_uc008bzj.2	20	26	+	10.8143	9.74e-05	0.253	CTTCCTG
1	mm10_knownGene_uc008xug.2	20	26	+	10.8143	9.74e-05	0.253	CTCCCTG
1	mm10_knownGene_uc012guh.1	20	26	+	10.8143	9.74e-05	0.253	CTTCCTG
1	mm10_knownGene_uc008zhw.2	14	20	-	10.8143	9.74e-05	0.253	CTTCCTG
1	mm10_knownGene_uc008stz.3	20	26	+	10.8143	9.74e-05	0.253	CTTCCTG
1	mm10_knownGene_uc008nxv.2	20	26	+	10.8143	9.74e-05	0.253	CTCCCTG
1	mm10_knownGene_uc009jwy.1	14	20	-	10.8143	9.74e-05	0.253	CTTCCTG
1	mm10_knownGene_uc007wrr.2	20	26	+	10.8143	9.74e-05	0.253	CTTCCTG
1	mm10_knownGene_uc007pen.1	20	26	+	10.8143	9.74e-05	0.253	CTCCCTG
1	mm10_knownGene_uc012dxj.1	20	26	+	10.8143	9.74e-05	0.253	CTCCCTG

1	mm10_knownGene_uc011ycw.1	14	20	-	10.8143	9.74e-05	0.253	CTTCCTG
1	mm10_knownGene_uc009fnl.2	14	20	-	10.8143	9.74e-05	0.253	CTCCCTG
1	mm10_knownGene_uc007lot.1	14	20	-	10.8143	9.74e-05	0.253	CTTCCTG
1	mm10_knownGene_uc029rqp.1	20	26	+	10.8143	9.74e-05	0.253	CTTCCTG
1	mm10_knownGene_uc007rvv.2	20	26	+	10.8143	9.74e-05	0.253	CTTCCTG
1	mm10_knownGene_uc008jep.1	20	26	+	10.8143	9.74e-05	0.253	CTCCCTG
1	mm10_knownGene_uc007kys.2	14	20	-	10.8143	9.74e-05	0.253	CTTCCTG
1	mm10_knownGene_uc007wrp.2	20	26	+	10.8143	9.74e-05	0.253	CTTCCTG
1	mm10_knownGene_uc007lou.1	14	20	-	10.8143	9.74e-05	0.253	CTTCCTG
1	mm10_knownGene_uc009haq.1	20	26	+	10.8143	9.74e-05	0.253	CTCCCTG
1	mm10_knownGene_uc008zhx.2	14	20	-	10.8143	9.74e-05	0.253	CTTCCTG
1	mm10_knownGene_uc011yfl.1	14	20	-	10.8143	9.74e-05	0.253	CTTCCTG
1	mm10_knownGene_uc007qkn.1	14	20	-	10.8143	9.74e-05	0.253	CTTCCTG
1	mm10_knownGene_uc008bzk.2	20	26	+	10.8143	9.74e-05	0.253	CTTCCTG
1	mm10_knownGene_uc057arj.1	14	20	-	10.8143	9.74e-05	0.253	CTTCCTG
1	mm10_knownGene_uc057ark.1	14	20	-	10.8143	9.74e-05	0.253	CTTCCTG
1	mm10_knownGene_uc009pwm.2	20	26	+	10.8143	9.74e-05	0.253	CTTCCTG
1	mm10_knownGene_uc029xhd.2	20	26	+	10.8143	9.74e-05	0.253	CTTCCTG
1	mm10_knownGene_uc007wro.2	20	26	+	10.8143	9.74e-05	0.253	CTTCCTG
1	mm10_knownGene_uc007kyr.2	14	20	-	10.8143	9.74e-05	0.253	CTTCCTG
1	mm10_knownGene_uc012apj.2	20	26	+	10.8143	9.74e-05	0.253	CTTCCTG
1	mm10_knownGene_uc057arl.1	14	20	-	10.8143	9.74e-05	0.253	CTTCCTG
1	mm10_knownGene_uc029uvl.1	20	26	+	10.8143	9.74e-05	0.253	CTTCCTG
1	mm10_knownGene_uc011ycv.1	14	20	-	10.8143	9.74e-05	0.253	CTTCCTG
1	mm10_knownGene_uc007wrq.2	20	26	+	10.8143	9.74e-05	0.253	CTTCCTG
1	mm10_knownGene_uc008bzi.2	20	26	+	10.8143	9.74e-05	0.253	CTTCCTG
1	mm10_knownGene_uc012fxj.1	14	20	-	10.8143	9.74e-05	0.253	CTCCCTG
1	mm10_knownGene_uc008xfr.2	20	26	+	10.8143	9.74e-05	0.253	CTTCCTG
1	mm10_knownGene_uc011zvw.2	20	26	+	10.8143	9.74e-05	0.253	CTTCCTG
1	mm10_knownGene_uc009jwz.1	14	20	-	10.8143	9.74e-05	0.253	CTTCCTG
1	mm10_knownGene_uc033ibs.1	15	21	-	10.8143	9.74e-05	0.253	CTCCCTG

1	mm10_knownGene_uc008eiz.1	21	27	+	10.8143	9.74e-05	0.253	CTTCCTG
1	mm10_knownGene_uc007eyv.2	15	21	-	10.8143	9.74e-05	0.253	CTCCCTG
1	mm10_knownGene_uc007eyu.2	15	21	-	10.8143	9.74e-05	0.253	CTCCCTG
1	mm10_knownGene_uc008sqk.1	15	21	-	10.8143	9.74e-05	0.253	CTCCCTG
1	mm10_knownGene_uc009pwu.1	15	21	-	10.8143	9.74e-05	0.253	CTTCCTG
1	mm10_knownGene_uc009hpv.1	21	27	+	10.8143	9.74e-05	0.253	CTTCCTG
1	mm10_knownGene_uc007wor.1	21	27	+	10.8143	9.74e-05	0.253	CTTCCTG
1	mm10_knownGene_uc008eja.1	21	27	+	10.8143	9.74e-05	0.253	CTTCCTG
1	mm10_knownGene_uc033fwy.1	21	27	+	10.8143	9.74e-05	0.253	CTTCCTG
1	mm10_knownGene_uc007wia.1	15	21	-	10.8143	9.74e-05	0.253	CTTCCTG
1	mm10_knownGene_uc009ass.1	15	21	-	10.8143	9.74e-05	0.253	CTCCCTG
1	mm10_knownGene_uc008vrc.1	21	27	+	10.8143	9.74e-05	0.253	CTTCCTG
1	mm10_knownGene_uc007cco.1	21	27	+	10.8143	9.74e-05	0.253	CTTCCTG
1	mm10_knownGene_uc007elg.1	15	21	-	10.8143	9.74e-05	0.253	CTTCCTG
1	mm10_knownGene_uc012ejd.1	21	27	+	10.8143	9.74e-05	0.253	CTCCCTG
1	mm10_knownGene_uc033idy.1	15	21	-	10.8143	9.74e-05	0.253	CTCCCTG
1	mm10_knownGene_uc029wna.1	21	27	+	10.8143	9.74e-05	0.253	CTTCCTG
1	mm10_knownGene_uc033gwv.1	15	21	-	10.8143	9.74e-05	0.253	CTCCCTG
1	mm10_knownGene_uc033idx.1	15	21	-	10.8143	9.74e-05	0.253	CTCCCTG
1	mm10_knownGene_uc011yip.1	21	27	+	10.8143	9.74e-05	0.253	CTTCCTG
1	mm10_knownGene_uc009kzi.1	21	27	+	10.8143	9.74e-05	0.253	CTTCCTG
1	mm10_knownGene_uc029tzw.1	15	21	-	10.8143	9.74e-05	0.253	CTTCCTG
1	mm10_knownGene_uc007hjw.1	21	27	+	10.8143	9.74e-05	0.253	CTCCCTG
1	mm10_knownGene_uc012hls.1	15	21	-	10.8143	9.74e-05	0.253	CTTCCTG
1	mm10_knownGene_uc009nlf.1	21	27	+	10.8143	9.74e-05	0.253	CTTCCTG
1	mm10_knownGene_uc029tmd.1	21	27	+	10.8143	9.74e-05	0.253	CTTCCTG
1	mm10_knownGene_uc008fza.1	15	21	-	10.8143	9.74e-05	0.253	CTCCCTG
1	mm10_knownGene_uc029vkz.1	15	21	-	10.8143	9.74e-05	0.253	CTCCCTG
1	mm10_knownGene_uc008mai.2	22	28	+	10.8143	9.74e-05	0.253	CTCCCTG
1	mm10_knownGene_uc009ssk.2	16	22	-	10.8143	9.74e-05	0.253	CTTCCTG
1	mm10_knownGene_uc009ssm.2	16	22	-	10.8143	9.74e-05	0.253	CTTCCTG

1	mm10_knownGene_uc007qko.2	22	28	+	10.8143	9.74e-05	0.253	CTTCCTG
1	mm10_knownGene_uc008mim.1	16	22	-	10.8143	9.74e-05	0.253	CTCCCTG
1	mm10_knownGene_uc029qxl.1	22	28	+	10.8143	9.74e-05	0.253	CTTCCTG
1	mm10_knownGene_uc009hze.1	16	22	-	10.8143	9.74e-05	0.253	CTCCCTG
1	mm10_knownGene_uc008jam.2	16	22	-	10.8143	9.74e-05	0.253	CTCCCTG
1	mm10_knownGene_uc007mfj.1	22	28	+	10.8143	9.74e-05	0.253	CTCCCTG
1	mm10_knownGene_uc008oao.2	22	28	+	10.8143	9.74e-05	0.253	CTCCCTG
1	mm10_knownGene_uc007uan.2	22	28	+	10.8143	9.74e-05	0.253	CTTCCTG
1	mm10_knownGene_uc009ssn.2	16	22	-	10.8143	9.74e-05	0.253	CTTCCTG
1	mm10_knownGene_uc011xng.1	16	22	-	10.8143	9.74e-05	0.253	CTTCCTG
1	mm10_knownGene_uc033inw.1	22	28	+	10.8143	9.74e-05	0.253	CTTCCTG
1	mm10_knownGene_uc029rfu.1	22	28	+	10.8143	9.74e-05	0.253	CTCCCTG
1	mm10_knownGene_uc007gsw.2	22	28	+	10.8143	9.74e-05	0.253	CTTCCTG
1	mm10_knownGene_uc007dyn.2	22	28	+	10.8143	9.74e-05	0.253	CTCCCTG
1	mm10_knownGene_uc009qur.2	16	22	-	10.8143	9.74e-05	0.253	CTTCCTG
1	mm10_knownGene_uc029wiw.1	16	22	-	10.8143	9.74e-05	0.253	CTCCCTG
1	mm10_knownGene_uc007eam.2	16	22	-	10.8143	9.74e-05	0.253	CTTCCTG
1	mm10_knownGene_uc009mhf.1	22	28	+	10.8143	9.74e-05	0.253	CTTCCTG
1	mm10_knownGene_uc007ipa.1	22	28	+	10.8143	9.74e-05	0.253	CTTCCTG
1	mm10_knownGene_uc008pyj.1	22	28	+	10.8143	9.74e-05	0.253	CTCCCTG
1	mm10_knownGene_uc008fdi.1	22	28	+	10.8143	9.74e-05	0.253	CTTCCTG
1	mm10_knownGene_uc007fhc.1	22	28	+	10.8143	9.74e-05	0.253	CTTCCTG
1	mm10_knownGene_uc007knw.1	16	22	-	10.8143	9.74e-05	0.253	CTCCCTG
1	mm10_knownGene_uc008srn.2	22	28	+	10.8143	9.74e-05	0.253	CTTCCTG
1	mm10_knownGene_uc007mnl.1	16	22	-	10.8143	9.74e-05	0.253	CTTCCTG
1	mm10_knownGene_uc009qen.2	16	22	-	10.8143	9.74e-05	0.253	CTTCCTG
1	mm10_knownGene_uc009qem.2	16	22	-	10.8143	9.74e-05	0.253	CTTCCTG
1	mm10_knownGene_uc029vnr.1	16	22	-	10.8143	9.74e-05	0.253	CTCCCTG
1	mm10_knownGene_uc009ssj.2	16	22	-	10.8143	9.74e-05	0.253	CTTCCTG
1	mm10_knownGene_uc008azo.2	22	28	+	10.8143	9.74e-05	0.253	CTTCCTG
1	mm10_knownGene_uc008hgs.1	22	28	+	10.8143	9.74e-05	0.253	CTTCCTG

1	mm10_knownGene_uc007ipb.1	22	28	+	10.8143	9.74e-05	0.253	CTTCCTG
1	mm10_knownGene_uc009ssl.2	16	22	-	10.8143	9.74e-05	0.253	CTTCCTG
1	mm10_knownGene_uc009gln.2	17	23	-	10.8143	9.74e-05	0.253	CTTCCTG
1	mm10_knownGene_uc007neh.1	17	23	-	10.8143	9.74e-05	0.253	CTCCCTG
1	mm10_knownGene_uc009min.2	23	29	+	10.8143	9.74e-05	0.253	CTCCCTG
1	mm10_knownGene_uc007tuz.3	23	29	+	10.8143	9.74e-05	0.253	CTCCCTG
1	mm10_knownGene_uc008lem.2	17	23	-	10.8143	9.74e-05	0.253	CTCCCTG
1	mm10_knownGene_uc029tef.1	17	23	-	10.8143	9.74e-05	0.253	CTTCCTG
1	mm10_knownGene_uc007hbz.2	17	23	-	10.8143	9.74e-05	0.253	CTTCCTG
1	mm10_knownGene_uc012bym.1	23	29	+	10.8143	9.74e-05	0.253	CTTCCTG
1	mm10_knownGene_uc009nqd.1	23	29	+	10.8143	9.74e-05	0.253	CTTCCTG
1	mm10_knownGene_uc008wcz.1	17	23	-	10.8143	9.74e-05	0.253	CTTCCTG
1	mm10_knownGene_uc007icg.2	17	23	-	10.8143	9.74e-05	0.253	CTTCCTG
1	mm10_knownGene_uc029vdz.1	23	29	+	10.8143	9.74e-05	0.253	CTTCCTG
1	mm10_knownGene_uc009hah.2	17	23	-	10.8143	9.74e-05	0.253	CTTCCTG
1	mm10_knownGene_uc009glm.2	17	23	-	10.8143	9.74e-05	0.253	CTTCCTG
1	mm10_knownGene_uc009nqe.1	23	29	+	10.8143	9.74e-05	0.253	CTTCCTG
1	mm10_knownGene_uc057brw.1	23	29	+	10.8143	9.74e-05	0.253	CTCCCTG
1	mm10_knownGene_uc008agl.3	23	29	+	10.8143	9.74e-05	0.253	CTCCCTG
1	mm10_knownGene_uc008xfe.1	23	29	+	10.8143	9.74e-05	0.253	CTCCCTG
1	mm10_knownGene_uc007gps.1	17	23	-	10.8143	9.74e-05	0.253	CTTCCTG
1	mm10_knownGene_uc007icf.2	17	23	-	10.8143	9.74e-05	0.253	CTTCCTG
1	mm10_knownGene_uc033fpd.1	23	29	+	10.8143	9.74e-05	0.253	CTTCCTG
1	mm10_knownGene_uc008tpf.2	17	23	-	10.8143	9.74e-05	0.253	CTTCCTG
1	mm10_knownGene_uc007gli.1	17	23	-	10.8143	9.74e-05	0.253	CTTCCTG
1	mm10_knownGene_uc009ndg.1	23	29	+	10.8143	9.74e-05	0.253	CTCCCTG
1	mm10_knownGene_uc008kgk.2	23	29	+	10.8143	9.74e-05	0.253	CTTCCTG
1	mm10_knownGene_uc007pyg.1	17	23	-	10.8143	9.74e-05	0.253	CTCCCTG
1	mm10_knownGene_uc007dsn.2	17	23	-	10.8143	9.74e-05	0.253	CTTCCTG
1	mm10_knownGene_uc056yrg.1	23	29	+	10.8143	9.74e-05	0.253	CTTCCTG
1	mm10_knownGene_uc007xgo.2	23	29	+	10.8143	9.74e-05	0.253	CTCCCTG

1	mm10_knownGene_uc008yjm.1	23	29	+	10.8143	9.74e-05	0.253	CTTCCTG
1	mm10_knownGene_uc033hug.1	23	29	+	10.8143	9.74e-05	0.253	CTCCCTG
1	mm10_knownGene_uc033gbw.1	23	29	+	10.8143	9.74e-05	0.253	CTTCCTG
1	mm10_knownGene_uc008hxd.2	17	23	-	10.8143	9.74e-05	0.253	CTCCCTG
1	mm10_knownGene_uc008hxc.2	17	23	-	10.8143	9.74e-05	0.253	CTCCCTG
1	mm10_knownGene_uc007ecg.1	23	29	+	10.8143	9.74e-05	0.253	CTTCCTG
1	mm10_knownGene_uc008faa.2	23	29	+	10.8143	9.74e-05	0.253	CTTCCTG
1	mm10_knownGene_uc029xns.1	17	23	-	10.8143	9.74e-05	0.253	CTCCCTG
1	mm10_knownGene_uc029sye.1	17	23	-	10.8143	9.74e-05	0.253	CTTCCTG
1	mm10_knownGene_uc029ute.1	23	29	+	10.8143	9.74e-05	0.253	CTTCCTG
1	mm10_knownGene_uc009nqf.1	23	29	+	10.8143	9.74e-05	0.253	CTTCCTG
1	mm10_knownGene_uc008ngz.1	17	23	-	10.8143	9.74e-05	0.253	CTCCCTG
1	mm10_knownGene_uc008qrg.2	23	29	+	10.8143	9.74e-05	0.253	CTTCCTG
1	mm10_knownGene_uc009gll.2	17	23	-	10.8143	9.74e-05	0.253	CTTCCTG
1	mm10_knownGene_uc007dat.1	17	23	-	10.8143	9.74e-05	0.253	CTTCCTG
1	mm10_knownGene_uc007oon.2	17	23	-	10.8143	9.74e-05	0.253	CTTCCTG
1	mm10_knownGene_uc007qcr.2	23	29	+	10.8143	9.74e-05	0.253	CTTCCTG
1	mm10_knownGene_uc029usb.1	23	29	+	10.8143	9.74e-05	0.253	CTTCCTG
1	mm10_knownGene_uc008pmn.3	23	29	+	10.8143	9.74e-05	0.253	CTCCCTG
1	mm10_knownGene_uc029tnd.1	23	29	+	10.8143	9.74e-05	0.253	CTTCCTG
1	mm10_knownGene_uc029tgf.1	24	30	+	10.8143	9.74e-05	0.253	CTCCCTG
1	mm10_knownGene_uc009mjx.2	18	24	-	10.8143	9.74e-05	0.253	CTCCCTG
1	mm10_knownGene_uc033ibp.1	24	30	+	10.8143	9.74e-05	0.253	CTTCCTG
1	mm10_knownGene_uc012hha.1	18	24	-	10.8143	9.74e-05	0.253	CTCCCTG
1	mm10_knownGene_uc008she.1	18	24	-	10.8143	9.74e-05	0.253	CTCCCTG
1	mm10_knownGene_uc008ulz.2	24	30	+	10.8143	9.74e-05	0.253	CTTCCTG
1	mm10_knownGene_uc029ukd.1	24	30	+	10.8143	9.74e-05	0.253	CTCCCTG
1	mm10_knownGene_uc008edn.2	18	24	-	10.8143	9.74e-05	0.253	CTCCCTG
1	mm10_knownGene_uc008ods.1	18	24	-	10.8143	9.74e-05	0.253	CTCCCTG
1	mm10_knownGene_uc029smk.1	18	24	-	10.8143	9.74e-05	0.253	CTTCCTG
1	mm10_knownGene_uc007nbt.2	24	30	+	10.8143	9.74e-05	0.253	CTCCCTG

1	mm10_knownGene_uc029srv.1	18	24	-	10.8143	9.74e-05	0.253	CTTCCTG
1	mm10_knownGene_uc008sjs.2	24	30	+	10.8143	9.74e-05	0.253	CTTCCTG
1	mm10_knownGene_uc008ord.4	24	30	+	10.8143	9.74e-05	0.253	CTCCCTG
1	mm10_knownGene_uc009hte.2	24	30	+	10.8143	9.74e-05	0.253	CTTCCTG
1	mm10_knownGene_uc009ghm.2	24	30	+	10.8143	9.74e-05	0.253	CTTCCTG
1	mm10_knownGene_uc008uly.2	24	30	+	10.8143	9.74e-05	0.253	CTTCCTG
1	mm10_knownGene_uc007nbv.1	24	30	+	10.8143	9.74e-05	0.253	CTCCCTG
1	mm10_knownGene_uc007nbu.2	24	30	+	10.8143	9.74e-05	0.253	CTCCCTG
1	mm10_knownGene_uc009eex.2	18	24	-	10.8143	9.74e-05	0.253	CTCCCTG
1	mm10_knownGene_uc007nbs.2	24	30	+	10.8143	9.74e-05	0.253	CTCCCTG
1	mm10_knownGene_uc007iyw.2	24	30	+	10.8143	9.74e-05	0.253	CTCCCTG
1	mm10_knownGene_uc008ore.4	24	30	+	10.8143	9.74e-05	0.253	CTCCCTG
1	mm10_knownGene_uc008sfy.2	24	30	+	10.8143	9.74e-05	0.253	CTTCCTG
1	mm10_knownGene_uc008edp.2	18	24	-	10.8143	9.74e-05	0.253	CTCCCTG
1	mm10_knownGene_uc007umi.2	24	30	+	10.8143	9.74e-05	0.253	CTCCCTG
1	mm10_knownGene_uc012dot.1	18	24	-	10.8143	9.74e-05	0.253	CTCCCTG
1	mm10_knownGene_uc009cla.2	18	24	-	10.8143	9.74e-05	0.253	CTTCCTG
1	mm10_knownGene_uc007ctn.1	24	30	+	10.8143	9.74e-05	0.253	CTTCCTG
1	mm10_knownGene_uc007uuq.1	24	30	+	10.8143	9.74e-05	0.253	CTTCCTG
1	mm10_knownGene_uc029tgi.1	18	24	-	10.8143	9.74e-05	0.253	CTCCCTG
1	mm10_knownGene_uc007upc.1	18	24	-	10.8143	9.74e-05	0.253	CTTCCTG
1	mm10_knownGene_uc008wsm.2	18	24	-	10.8143	9.74e-05	0.253	CTCCCTG
1	mm10_knownGene_uc029sle.1	24	30	+	10.8143	9.74e-05	0.253	CTCCCTG
1	mm10_knownGene_uc007ohb.1	24	30	+	10.8143	9.74e-05	0.253	CTCCCTG
1	mm10_knownGene_uc008sfz.1	24	30	+	10.8143	9.74e-05	0.253	CTTCCTG
1	mm10_knownGene_uc008ulx.2	24	30	+	10.8143	9.74e-05	0.253	CTTCCTG
1	mm10_knownGene_uc008vrh.1	18	24	-	10.8143	9.74e-05	0.253	CTCCCTG
1	mm10_knownGene_uc008ulw.2	24	30	+	10.8143	9.74e-05	0.253	CTTCCTG
1	mm10_knownGene_uc009hrn.2	24	30	+	10.8143	9.74e-05	0.253	CTTCCTG
1	mm10_knownGene_uc008dvt.2	18	24	-	10.8143	9.74e-05	0.253	CTTCCTG
1	mm10_knownGene_uc008sjr.2	24	30	+	10.8143	9.74e-05	0.253	CTTCCTG

1	mm10_knownGene_uc007xgj.2	18	24	-	10.8143	9.74e-05	0.253	CTTCCTG
1	mm10_knownGene_uc008unk.1	18	24	-	10.8143	9.74e-05	0.253	CTTCCTG
1	mm10_knownGene_uc008cnu.1	18	24	-	10.8143	9.74e-05	0.253	CTCCCTG
1	mm10_knownGene_uc012aqi.2	24	30	+	10.8143	9.74e-05	0.253	CTTCCTG
1	mm10_knownGene_uc008blx.2	18	24	-	10.8143	9.74e-05	0.253	CTTCCTG
1	mm10_knownGene_uc029uke.1	24	30	+	10.8143	9.74e-05	0.253	CTCCCTG
1	mm10_knownGene_uc029vtk.1	24	30	+	10.8143	9.74e-05	0.253	CTTCCTG
1	mm10_knownGene_uc029ulh.1	19	25	-	10.8143	9.74e-05	0.253	CTCCCTG
1	mm10_knownGene_uc009fle.2	19	25	-	10.8143	9.74e-05	0.253	CTCCCTG
1	mm10_knownGene_uc029tri.1	25	31	+	10.8143	9.74e-05	0.253	CTTCCTG
1	mm10_knownGene_uc029uwr.1	25	31	+	10.8143	9.74e-05	0.253	CTTCCTG
1	mm10_knownGene_uc007gsc.1	25	31	+	10.8143	9.74e-05	0.253	CTTCCTG
1	mm10_knownGene_uc033iww.1	19	25	-	10.8143	9.74e-05	0.253	CTCCCTG
1	mm10_knownGene_uc008fei.1	25	31	+	10.8143	9.74e-05	0.253	CTTCCTG
1	mm10_knownGene_uc007ntg.2	25	31	+	10.8143	9.74e-05	0.253	CTTCCTG
1	mm10_knownGene_uc008ayo.2	25	31	+	10.8143	9.74e-05	0.253	CTTCCTG
1	mm10_knownGene_uc008fej.1	25	31	+	10.8143	9.74e-05	0.253	CTTCCTG
1	mm10_knownGene_uc033imn.1	19	25	-	10.8143	9.74e-05	0.253	CTTCCTG
1	mm10_knownGene_uc007pbv.1	19	25	-	10.8143	9.74e-05	0.253	CTCCCTG
1	mm10_knownGene_uc007pby.1	19	25	-	10.8143	9.74e-05	0.253	CTCCCTG
1	mm10_knownGene_uc007mqq.2	25	31	+	10.8143	9.74e-05	0.253	CTTCCTG
1	mm10_knownGene_uc029wvu.1	19	25	-	10.8143	9.74e-05	0.253	CTTCCTG
1	mm10_knownGene_uc007ntj.2	25	31	+	10.8143	9.74e-05	0.253	CTTCCTG
1	mm10_knownGene_uc029rwx.1	19	25	-	10.8143	9.74e-05	0.253	CTTCCTG
1	mm10_knownGene_uc009mye.2	25	31	+	10.8143	9.74e-05	0.253	CTTCCTG
1	mm10_knownGene_uc009flf.2	19	25	-	10.8143	9.74e-05	0.253	CTCCCTG
1	mm10_knownGene_uc007wjh.1	25	31	+	10.8143	9.74e-05	0.253	CTCCCTG
1	mm10_knownGene_uc011yng.2	25	31	+	10.8143	9.74e-05	0.253	CTTCCTG
1	mm10_knownGene_uc033gqs.1	25	31	+	10.8143	9.74e-05	0.253	CTTCCTG
1	mm10_knownGene_uc008dag.1	25	31	+	10.8143	9.74e-05	0.253	CTCCCTG
1	mm10_knownGene_uc008yel.1	25	31	+	10.8143	9.74e-05	0.253	CTTCCTG

1	mm10_knownGene_uc009sts.2	19	25	-	10.8143	9.74e-05	0.253	CTCCCTG
1	mm10_knownGene_uc007wiz.2	25	31	+	10.8143	9.74e-05	0.253	CTCCCTG
1	mm10_knownGene_uc008mwn.1	25	31	+	10.8143	9.74e-05	0.253	CTTCCTG
1	mm10_knownGene_uc012fba.1	19	25	-	10.8143	9.74e-05	0.253	CTCCCTG
1	mm10_knownGene_uc007ybq.1	25	31	+	10.8143	9.74e-05	0.253	CTCCCTG
1	mm10_knownGene_uc057bew.1	19	25	-	10.8143	9.74e-05	0.253	CTTCCTG
1	mm10_knownGene_uc009tnn.2	19	25	-	10.8143	9.74e-05	0.253	CTCCCTG
1	mm10_knownGene_uc007qbg.1	25	31	+	10.8143	9.74e-05	0.253	CTCCCTG
1	mm10_knownGene_uc009uzn.2	19	25	-	10.8143	9.74e-05	0.253	CTCCCTG
1	mm10_knownGene_uc029vnm.1	19	25	-	10.8143	9.74e-05	0.253	CTCCCTG
1	mm10_knownGene_uc029rtq.1	25	31	+	10.8143	9.74e-05	0.253	CTTCCTG
1	mm10_knownGene_uc056zzw.1	19	25	-	10.8143	9.74e-05	0.253	CTCCCTG
1	mm10_knownGene_uc009cpd.1	25	31	+	10.8143	9.74e-05	0.253	CTCCCTG
1	mm10_knownGene_uc009stu.2	19	25	-	10.8143	9.74e-05	0.253	CTCCCTG
1	mm10_knownGene_uc009uzm.2	19	25	-	10.8143	9.74e-05	0.253	CTCCCTG
1	mm10_knownGene_uc007ckt.3	19	25	-	10.8143	9.74e-05	0.253	CTTCCTG
1	mm10_knownGene_uc009gnr.2	19	25	-	10.8143	9.74e-05	0.253	CTCCCTG
1	mm10_knownGene_uc008zep.1	19	25	-	10.8143	9.74e-05	0.253	CTCCCTG
1	mm10_knownGene_uc011zdf.1	25	31	+	10.8143	9.74e-05	0.253	CTTCCTG
1	mm10_knownGene_uc007ybp.1	25	31	+	10.8143	9.74e-05	0.253	CTCCCTG
1	mm10_knownGene_uc011ynh.2	25	31	+	10.8143	9.74e-05	0.253	CTTCCTG
1	mm10_knownGene_uc009gio.2	25	31	+	10.8143	9.74e-05	0.253	CTTCCTG
1	mm10_knownGene_uc007nti.2	25	31	+	10.8143	9.74e-05	0.253	CTTCCTG
1	mm10_knownGene_uc008gql.1	19	25	-	10.8143	9.74e-05	0.253	CTTCCTG
1	mm10_knownGene_uc007nth.2	25	31	+	10.8143	9.74e-05	0.253	CTTCCTG
1	mm10_knownGene_uc009myd.2	25	31	+	10.8143	9.74e-05	0.253	CTTCCTG
1	mm10_knownGene_uc009lss.2	25	31	+	10.8143	9.74e-05	0.253	CTCCCTG
1	mm10_knownGene_uc007awm.2	25	31	+	10.8143	9.74e-05	0.253	CTCCCTG
1	mm10_knownGene_uc008lkm.1	25	31	+	10.8143	9.74e-05	0.253	CTTCCTG
1	mm10_knownGene_uc009stt.2	19	25	-	10.8143	9.74e-05	0.253	CTCCCTG
1	mm10_knownGene_uc008ncc.1	25	31	+	10.8143	9.74e-05	0.253	CTTCCTG

1	mm10_knownGene_uc008feh.1	25	31	+	10.8143	9.74e-05	0.253	CTTCCTG
1	mm10_knownGene_uc007pbw.1	19	25	-	10.8143	9.74e-05	0.253	CTCCCTG
1	mm10_knownGene_uc007awn.2	25	31	+	10.8143	9.74e-05	0.253	CTCCCTG
1	mm10_knownGene_uc007pbx.1	19	25	-	10.8143	9.74e-05	0.253	CTCCCTG
1	mm10_knownGene_uc007awl.2	25	31	+	10.8143	9.74e-05	0.253	CTCCCTG
1	mm10_knownGene_uc057cgf.1	25	31	+	10.8143	9.74e-05	0.253	CTCCCTG
1	mm10_knownGene_uc008mlu.1	26	32	+	10.8143	9.74e-05	0.253	CTCCCTG
1	mm10_knownGene_uc009arv.2	20	26	-	10.8143	9.74e-05	0.253	CTTCCTG
1	mm10_knownGene_uc008crm.1	26	32	+	10.8143	9.74e-05	0.253	CTCCCTG
1	mm10_knownGene_uc008tzc.1	26	32	+	10.8143	9.74e-05	0.253	CTCCCTG
1	mm10_knownGene_uc012gfi.1	20	26	-	10.8143	9.74e-05	0.253	CTCCCTG
1	mm10_knownGene_uc008bmf.1	26	32	+	10.8143	9.74e-05	0.253	CTTCCTG
1	mm10_knownGene_uc029uvx.1	26	32	+	10.8143	9.74e-05	0.253	CTTCCTG
1	mm10_knownGene_uc009bce.1	26	32	+	10.8143	9.74e-05	0.253	CTTCCTG
1	mm10_knownGene_uc009dlj.2	26	32	+	10.8143	9.74e-05	0.253	CTCCCTG
1	mm10_knownGene_uc008mns.1	20	26	-	10.8143	9.74e-05	0.253	CTTCCTG
1	mm10_knownGene_uc008ojg.1	20	26	-	10.8143	9.74e-05	0.253	CTCCCTG
1	mm10_knownGene_uc009mbr.2	20	26	-	10.8143	9.74e-05	0.253	CTCCCTG
1	mm10_knownGene_uc057bzo.1	20	26	-	10.8143	9.74e-05	0.253	CTTCCTG
1	mm10_knownGene_uc007sgx.1	20	26	-	10.8143	9.74e-05	0.253	CTTCCTG
1	mm10_knownGene_uc009lpf.1	26	32	+	10.8143	9.74e-05	0.253	CTTCCTG
1	mm10_knownGene_uc008sks.1	20	26	-	10.8143	9.74e-05	0.253	CTTCCTG
1	mm10_knownGene_uc029ujf.2	20	26	-	10.8143	9.74e-05	0.253	CTCCCTG
1	mm10_knownGene_uc029vrs.1	20	26	-	10.8143	9.74e-05	0.253	CTTCCTG
1	mm10_knownGene_uc012cmd.1	20	26	-	10.8143	9.74e-05	0.253	CTCCCTG
1	mm10_knownGene_uc009bax.2	26	32	+	10.8143	9.74e-05	0.253	CTTCCTG
1	mm10_knownGene_uc008utx.1	26	32	+	10.8143	9.74e-05	0.253	CTCCCTG
1	mm10_knownGene_uc009baw.2	26	32	+	10.8143	9.74e-05	0.253	CTTCCTG
1	mm10_knownGene_uc008oij.2	20	26	-	10.8143	9.74e-05	0.253	CTCCCTG
1	mm10_knownGene_uc009kbf.1	26	32	+	10.8143	9.74e-05	0.253	CTCCCTG
1	mm10_knownGene_uc007cxy.1	26	32	+	10.8143	9.74e-05	0.253	CTTCCTG

1	mm10_knownGene_uc008cos.1	26	32	+	10.8143	9.74e-05	0.253	CTCCCTG
1	mm10_knownGene_uc007lec.1	26	32	+	10.8143	9.74e-05	0.253	CTCCCTG
1	mm10_knownGene_uc033jum.1	26	32	+	10.8143	9.74e-05	0.253	CTTCCTG
1	mm10_knownGene_uc033gys.1	20	26	-	10.8143	9.74e-05	0.253	CTCCCTG
1	mm10_knownGene_uc009bcg.1	26	32	+	10.8143	9.74e-05	0.253	CTTCCTG
1	mm10_knownGene_uc007uob.2	26	32	+	10.8143	9.74e-05	0.253	CTCCCTG
1	mm10_knownGene_uc009iqw.1	20	26	-	10.8143	9.74e-05	0.253	CTTCCTG
1	mm10_knownGene_uc009bcf.1	26	32	+	10.8143	9.74e-05	0.253	CTTCCTG
1	mm10_knownGene_uc008hga.2	26	32	+	10.8143	9.74e-05	0.253	CTCCCTG
1	mm10_knownGene_uc008eru.1	26	32	+	10.8143	9.74e-05	0.253	CTCCCTG
1	mm10_knownGene_uc008utw.1	26	32	+	10.8143	9.74e-05	0.253	CTCCCTG
1	mm10_knownGene_uc012bks.1	26	32	+	10.8143	9.74e-05	0.253	CTCCCTG
1	mm10_knownGene_uc007dgw.1	26	32	+	10.8143	9.74e-05	0.253	CTTCCTG
1	mm10_knownGene_uc008ypr.2	20	26	-	10.8143	9.74e-05	0.253	CTTCCTG
1	mm10_knownGene_uc008dls.1	20	26	-	10.8143	9.74e-05	0.253	CTTCCTG
1	mm10_knownGene_uc008apz.2	26	32	+	10.8143	9.74e-05	0.253	CTTCCTG
1	mm10_knownGene_uc012eqz.1	27	33	+	10.8143	9.74e-05	0.253	CTTCCTG
1	mm10_knownGene_uc012fjv.1	21	27	-	10.8143	9.74e-05	0.253	CTTCCTG
1	mm10_knownGene_uc029qnu.1	27	33	+	10.8143	9.74e-05	0.253	CTCCCTG
1	mm10_knownGene_uc008qvj.1	21	27	-	10.8143	9.74e-05	0.253	CTCCCTG
1	mm10_knownGene_uc008tzi.1	27	33	+	10.8143	9.74e-05	0.253	CTCCCTG
1	mm10_knownGene_uc007unn.1	27	33	+	10.8143	9.74e-05	0.253	CTCCCTG
1	mm10_knownGene_uc012egm.1	21	27	-	10.8143	9.74e-05	0.253	CTCCCTG
1	mm10_knownGene_uc012gru.1	21	27	-	10.8143	9.74e-05	0.253	CTCCCTG
1	mm10_knownGene_uc009grc.1	21	27	-	10.8143	9.74e-05	0.253	CTTCCTG
1	mm10_knownGene_uc056ykh.1	21	27	-	10.8143	9.74e-05	0.253	CTCCCTG
1	mm10_knownGene_uc029sbv.1	21	27	-	10.8143	9.74e-05	0.253	CTTCCTG
1	mm10_knownGene_uc009jjs.1	21	27	-	10.8143	9.74e-05	0.253	CTCCCTG
1	mm10_knownGene_uc009coz.2	21	27	-	10.8143	9.74e-05	0.253	CTCCCTG
1	mm10_knownGene_uc007oiu.2	27	33	+	10.8143	9.74e-05	0.253	CTCCCTG
1	mm10_knownGene_uc008hpo.1	21	27	-	10.8143	9.74e-05	0.253	CTCCCTG

1	mm10_knownGene_uc011zit.1	27	33	+	10.8143	9.74e-05	0.253	CTTCCTG
1	mm10_knownGene_uc029vyr.1	21	27	-	10.8143	9.74e-05	0.253	CTCCCTG
1	mm10_knownGene_uc009iwd.1	27	33	+	10.8143	9.74e-05	0.253	CTCCCTG
1	mm10_knownGene_uc008boo.2	27	33	+	10.8143	9.74e-05	0.253	CTCCCTG
1	mm10_knownGene_uc007mec.1	27	33	+	10.8143	9.74e-05	0.253	CTTCCTG
1	mm10_knownGene_uc012era.1	27	33	+	10.8143	9.74e-05	0.253	CTTCCTG
1	mm10_knownGene_uc057caq.1	27	33	+	10.8143	9.74e-05	0.253	CTCCCTG
1	mm10_knownGene_uc007ujt.1	21	27	-	10.8143	9.74e-05	0.253	CTTCCTG
1	mm10_knownGene_uc009aol.1	27	33	+	10.8143	9.74e-05	0.253	CTTCCTG
1	mm10_knownGene_uc009orh.1	21	27	-	10.8143	9.74e-05	0.253	CTTCCTG
1	mm10_knownGene_uc009dvu.2	21	27	-	10.8143	9.74e-05	0.253	CTCCCTG
1	mm10_knownGene_uc057car.1	27	33	+	10.8143	9.74e-05	0.253	CTCCCTG
1	mm10_knownGene_uc009ipt.1	27	33	+	10.8143	9.74e-05	0.253	CTTCCTG
1	mm10_knownGene_uc012fpt.1	28	34	+	10.8143	9.74e-05	0.253	CTCCCTG
1	mm10_knownGene_uc009hpu.1	28	34	+	10.8143	9.74e-05	0.253	CTCCCTG
1	mm10_knownGene_uc009qrl.2	28	34	+	10.8143	9.74e-05	0.253	CTTCCTG
1	mm10_knownGene_uc008nqi.1	28	34	+	10.8143	9.74e-05	0.253	CTTCCTG
1	mm10_knownGene_uc008tnd.1	22	28	-	10.8143	9.74e-05	0.253	CTCCCTG
1	mm10_knownGene_uc008gyw.2	22	28	-	10.8143	9.74e-05	0.253	CTCCCTG
1	mm10_knownGene_uc007frl.2	28	34	+	10.8143	9.74e-05	0.253	CTCCCTG
1	mm10_knownGene_uc029xiv.1	22	28	-	10.8143	9.74e-05	0.253	CTCCCTG
1	mm10_knownGene_uc009oyv.1	22	28	-	10.8143	9.74e-05	0.253	CTTCCTG
1	mm10_knownGene_uc009mta.2	28	34	+	10.8143	9.74e-05	0.253	CTTCCTG
1	mm10_knownGene_uc008wip.2	22	28	-	10.8143	9.74e-05	0.253	CTTCCTG
1	mm10_knownGene_uc007ytw.2	22	28	-	10.8143	9.74e-05	0.253	CTTCCTG
1	mm10_knownGene_uc007veq.2	22	28	-	10.8143	9.74e-05	0.253	CTCCCTG
1	mm10_knownGene_uc008chm.2	22	28	-	10.8143	9.74e-05	0.253	CTCCCTG
1	mm10_knownGene_uc009jiw.1	22	28	-	10.8143	9.74e-05	0.253	CTTCCTG
1	mm10_knownGene_uc007cbr.1	22	28	-	10.8143	9.74e-05	0.253	CTCCCTG
1	mm10_knownGene_uc012bnq.2	28	34	+	10.8143	9.74e-05	0.253	CTTCCTG
1	mm10_knownGene_uc009rho.1	22	28	-	10.8143	9.74e-05	0.253	CTTCCTG

1	mm10_knownGene_uc009rhl.1	22	28	-	10.8143	9.74e-05	0.253	CTTCCTG
1	mm10_knownGene_uc029trk.1	22	28	-	10.8143	9.74e-05	0.253	CTTCCTG
1	mm10_knownGene_uc007tzi.1	28	34	+	10.8143	9.74e-05	0.253	CTTCCTG
1	mm10_knownGene_uc009rhn.1	22	28	-	10.8143	9.74e-05	0.253	CTTCCTG
1	mm10_knownGene_uc009rhm.1	22	28	-	10.8143	9.74e-05	0.253	CTTCCTG
1	mm10_knownGene_uc007dyg.1	28	34	+	10.8143	9.74e-05	0.253	CTCCCTG
1	mm10_knownGene_uc008eyu.1	28	34	+	10.8143	9.74e-05	0.253	CTCCCTG
1	mm10_knownGene_uc007ixo.1	22	28	-	10.8143	9.74e-05	0.253	CTTCCTG
1	mm10_knownGene_uc008pui.1	28	34	+	10.8143	9.74e-05	0.253	CTTCCTG
1	mm10_knownGene_uc009bdq.1	28	34	+	10.8143	9.74e-05	0.253	CTTCCTG
1	mm10_knownGene_uc009etk.1	22	28	-	10.8143	9.74e-05	0.253	CTTCCTG
1	mm10_knownGene_uc008emx.1	22	28	-	10.8143	9.74e-05	0.253	CTTCCTG
1	mm10_knownGene_uc007ver.2	22	28	-	10.8143	9.74e-05	0.253	CTCCCTG
1	mm10_knownGene_uc011yde.2	29	35	+	10.8143	9.74e-05	0.253	CTCCCTG
1	mm10_knownGene_uc008qsl.3	29	35	+	10.8143	9.74e-05	0.253	CTTCCTG
1	mm10_knownGene_uc008nvu.1	23	29	-	10.8143	9.74e-05	0.253	CTTCCTG
1	mm10_knownGene_uc011zby.2	23	29	-	10.8143	9.74e-05	0.253	CTTCCTG
1	mm10_knownGene_uc033fjg.1	23	29	-	10.8143	9.74e-05	0.253	CTTCCTG
1	mm10_knownGene_uc011ydh.1	23	29	-	10.8143	9.74e-05	0.253	CTTCCTG
1	mm10_knownGene_uc033fzl.1	29	35	+	10.8143	9.74e-05	0.253	CTCCCTG
1	mm10_knownGene_uc009pqf.2	23	29	-	10.8143	9.74e-05	0.253	CTTCCTG
1	mm10_knownGene_uc009drm.1	29	35	+	10.8143	9.74e-05	0.253	CTTCCTG
1	mm10_knownGene_uc008fjh.1	23	29	-	10.8143	9.74e-05	0.253	CTCCCTG
1	mm10_knownGene_uc029tco.1	23	29	-	10.8143	9.74e-05	0.253	CTTCCTG
1	mm10_knownGene_uc008fmx.1	23	29	-	10.8143	9.74e-05	0.253	CTTCCTG
1	mm10_knownGene_uc029wxj.1	23	29	-	10.8143	9.74e-05	0.253	CTCCCTG
1	mm10_knownGene_uc008geo.2	29	35	+	10.8143	9.74e-05	0.253	CTTCCTG
1	mm10_knownGene_uc007eaz.1	29	35	+	10.8143	9.74e-05	0.253	CTTCCTG
1	mm10_knownGene_uc007geq.1	23	29	-	10.8143	9.74e-05	0.253	CTTCCTG
1	mm10_knownGene_uc007eyu.2	23	29	-	10.8143	9.74e-05	0.253	CTTCCTG
1	mm10_knownGene_uc033fwn.1	23	29	-	10.8143	9.74e-05	0.253	CTTCCTG

1	mm10_knownGene_uc008nvv.1	23	29	-	10.8143 9.74e-
05	0.253	CTTCCTG
1	mm10_knownGene_uc007mfz.1	29	35	+	10.8143 9.74e-
05	0.253	CTTCCTG
1	mm10_knownGene_u
